# Complexes of Usher proteins preassemble at the endoplasmic reticulum and are required for trafficking and ER homeostasis

**DOI:** 10.1242/dmm.014068

**Published:** 2014-03-13

**Authors:** Bernardo Blanco-Sánchez, Aurélie Clément, Javier Fierro, Philip Washbourne, Monte Westerfield

**Affiliations:** Institute of Neuroscience, University of Oregon, Eugene, OR 97403, USA.

**Keywords:** Harmonin, Cadherin23, Ift88, Myo7aa, Usher syndrome, Hair cell, Trafficking, ER stress, Zebrafish

## Abstract

Usher syndrome (USH), the leading cause of hereditary combined hearing and vision loss, is characterized by sensorineural deafness and progressive retinal degeneration. Mutations in several different genes produce USH, but the proximal cause of sensory cell death remains mysterious. We adapted a proximity ligation assay to analyze associations among three of the USH proteins, Cdh23, Harmonin and Myo7aa, and the microtubule-based transporter Ift88 in zebrafish inner ear mechanosensory hair cells. We found that the proteins are in close enough proximity to form complexes and that these complexes preassemble at the endoplasmic reticulum (ER). Defects in any one of the three USH proteins disrupt formation and trafficking of the complex and result in diminished levels of the other proteins, generalized trafficking defects and ER stress that triggers apoptosis. ER stress, thus, contributes to sensory hair cell loss and provides a new target to explore for protective therapies for USH.

## INTRODUCTION

Usher syndrome (USH) is a multigenic disease that affects 1 in 6000 people in the United States (Kimberling et al., 2010). Individuals with USH present with hearing loss, progressive retinal degeneration and sometimes balance dysfunction. There are three clinical types, USH1, USH2 and USH3, based on the age of onset and severity of auditory and visual symptoms, with USH1 being the most severe. Individuals with USH1 have profound congenital deafness, balance problems and relatively early-onset retinitis pigmentosa.

To date, 11 different genes (13 genetic loci) have been linked to USH (Bonnet and El-Amraoui, 2012; Jaworek et al., 2012; Puffenberger et al., 2012; Riazuddin et al., 2012). The known USH genes encode proteins with diverse functions, including transmembrane adhesion and signaling, scaffolding, and myosinmotor transport. *In vitro* binding assays have led to an emerging view that harmonin (USH1C) and whirlin (DFNB31, USH2D) act as scaffold proteins that assemble the Usher proteins into a multimolecular complex (Adato et al., 2005b; Boëda et al., 2002; Maerker et al., 2008; Reiners et al., 2006; Söllner et al., 2004; van Wijk et al., 2006). We recently showed that PDZD7 serves as a genetic modifier and an additional scaffold for Usher proteins (Ebermann et al., 2010). This multi-molecular-complex model could provide an explanation for how mutations in such a wide variety of different types of proteins can produce such similar symptoms; if the proteins work together in complexes, then defects in any one component protein might be expected to compromise function of the complex as a whole. In mechanosensory hair cells, the USH proteins have been shown to play structural and developmental roles in stereocilia and ribbon synapses (Lefèvre et al., 2008; McGee et al., 2006; Seiler et al., 2005; Söllner et al., 2004; van Wijk et al., 2006; Zallocchi et al., 2012). In the retina, USH proteins are associated with the connecting cilium, outer limiting membrane (Gosens et al., 2007) and ribbon synapses of photoreceptors (Kersten et al., 2010; Maerker et al., 2008; Overlack et al., 2008; Phillips et al., 2011). Defects in these proteins are known to disrupt function of mechanosensory hair cells and photoreceptors; however, the eventual cause of cell death in USH is unknown.

To examine potential interactions among the USH proteins *in situ*, we adapted a proximity ligation assay (Söderberg et al., 2006) to use on whole-mount zebrafish inner ear mechanosensory hair cells. We examined localization and proximity of one of each of the three different types of USH1 proteins: transmembrane (Cdh23), scaffold (Harmonin) and actin-based motor (Myo7a). A defect in any one of these proteins produces the severe form of USH (Bonnet and El-Amraoui, 2012), and recent studies have shown that they can form a ternary complex that interacts with membrane phospholipids *in vitro* (Bahloul et al., 2010). We examined the early stages of hair cell development and found that these three USH1 proteins are in close enough proximity to form a complex. We see that these interactions occur in close proximity to COPII proteins, indicating that the complex preassembles in the endoplasmic reticulum (ER) before it is trafficked to the Golgi. To characterize the trafficking, we examined Ift88, an intraflagellar transport protein (Pazour et al., 2002), and found that it, too, is associated with the complex of these USH1 and COPII proteins. These close interactions are disrupted by mutations in any one of the genes, the remaining proteins are diminished and mislocalized, and the cells develop generalized trafficking defects. Thus, our data demonstrate that the Cdh23, Harmonin, Myo7aa and Ift88 complex is functionally active during vesicular transport from the ER and acts as a newly identified trafficking regulator in the hair cell. Our results also show that defects in the complex produce ER stress that leads to apoptosis.

## RESULTS

### Mutations in different USH1 genes and in *Ift88* lead to severe structural mechanoreceptor defects

**Fig. 1:**
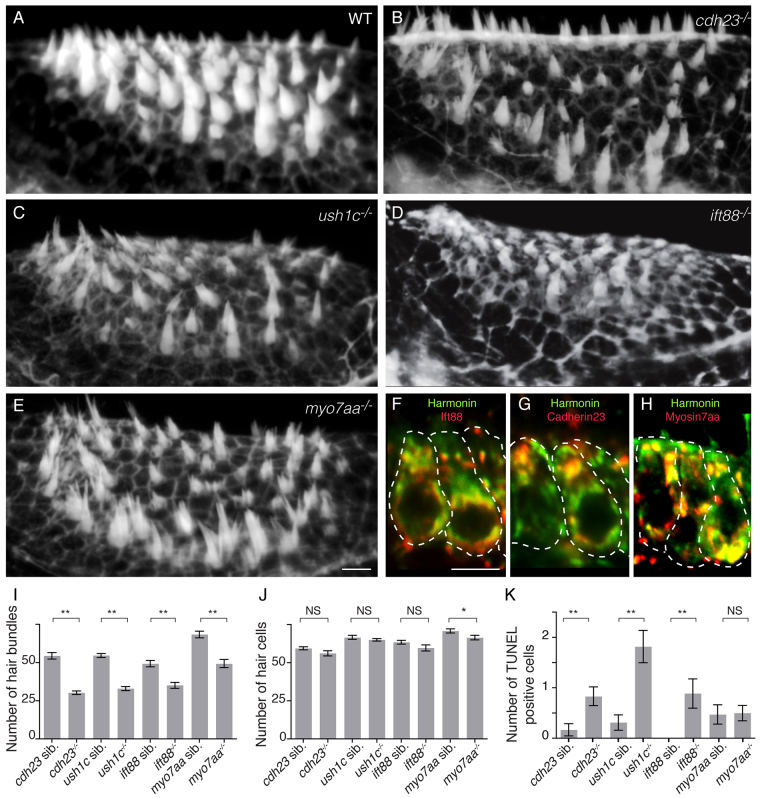
**Zebrafish *cdh23*, *ush1c*, *myo7aa* and *ift88* mutants have mechanoreceptor structural defects.** (A–E) Confocal projections of anterior maculae labeled with phalloidin in (A) wild-type (WT) sibling, (B) *cdh23^tj264a^*, (C) *ush1c^fh293^*, (D) *ift88^tz288b^* and (E) *myo7aa^ty220d^* mutants. (F–H) Confocal optical sections of double immunolabeling of (F) Harmonin (green) and Ift88 (red), (G) Harmonin (green) and Cdh23 (red), and (H) Harmonin (green) and Myo7aa (red). Individual hair cells are shown. (I) Quantification of total number of hair bundles. *n*≥6 analyzed anterior maculae. (J) Quantification of total number of hair cells. *n*≥5 analyzed anterior maculae. (K) Quantification of hair cell death in mutants and wild-type siblings. Number: average number of TUNEL-positive hair cells per macula. *n*≥16 analyzed anterior maculae. Student’s *t*-test: **P<0.01, *P<0.05. NS, non significant. Black lines represent ± s.e.m. Scale bars: 5 μm.

We examined the phenotypes resulting from mutations in three of the zebrafish USH1 genes, *cdh23*, *ush1c* and *myo7aa*, and in *ift88*, a gene that encodes an intraflagellar transport protein known to function in the development of inner ear mechanosensory hair cells (Jones et al., 2008; Kindt et al., 2012; Tsujikawa and Malicki, 2004). We previously showed that *ush1c* mutants have hearing and balance defects (Phillips et al., 2011), and other studies have shown similar phenotypes in *myo7aa* (Ernest et al., 2000) and *cdh23* (Söllner et al., 2004) zebrafish mutants. To characterize the cellular basis of these defects, we examined the number and the structure of hair cells and stereocilia in the anterior macula of the inner ear. All four mutants showed similar significant structural defects in mechanosensory hair cells, including bent and/or splayed stereocilia (Fig. 1B–E), consistent with the known requirements of USH1 proteins (Bonnet and El-Amraoui, 2012) and Ift88 (Jones et al., 2008) during hair bundle morphogenesis. Additionally, fewer hair cells (~65%) formed stereocilia in all four mutants (Fig. 1I) compared with controls, as previously reported for *ush1c* mutants (Phillips et al., 2011). We also observed an increase in apoptosis of hair cells in *cdh23* and *ift88* mutants, as well as in *ush1c* mutants (Fig. 1K), as we showed previously (Phillips et al., 2011). Despite these defects, the epithelial organization (supplementary material Fig. S1) and number of hair cells formed (Fig. 1J) were relatively normal in the mutants, indicating that initial specification of hair cell fates does not depend upon USH1 or Ift88 function.

**TRANSLATIONAL IMPACT**

**Clinical issue**

Usher syndrome is a recessively inherited disease of combined deafness and blindness that is characterized by the loss of sensory cells. Hearing impairment in people with Usher syndrome is usually apparent from birth, whereas vision loss is slow and progressive, and begins in the first or second decade of life. There is a great deal of clinical variability among patients with Usher syndrome and, to date, 11 genes that can cause this disease when mutated have been identified. These genes encode proteins that have a wide variety of functions and that can interact physically with each other to form macromolecular complexes. A better understanding of where in the cell these complexes assemble and how mutations in their component proteins cause cell death will enhance our understanding of the etiology of Usher syndrome, and could contribute to the development of effective treatments.

**Results**

In this paper, the authors describe the assembly of a complex of three known Usher proteins – the scaffold protein Harmonin, the transmembrane adhesion protein Cadherin23 and the actin-based motor Myosin7aa – and the intraflagellar transport protein Ift88, a newly identified interactor, in zebrafish inner ear mechanosensory hair cells. The authors report that mutations in any one of these proteins lead to similar structural defects in the mechanosensory cells. Then, using an *in vivo* proximity ligation assay, they demonstrate that the protein complex containing these four proteins assembles at the level of the endoplasmic reticulum (ER) in mechanosensory cells. Compromising the function of any member of the complex not only blocks the formation of the complex, but also results in severe trafficking defects, ER stress and cell death. Finally, they report that cell death is reduced by blocking the function of Cdk5, a factor that mediates ER-stress-induced apoptosis.

**Implications and future directions**

These findings show that ER stress is the underlying cause of sensory cell loss in Usher syndrome and provide a major advance for the field by identifying, for the first time, the proximal cause of sensory cell loss in this disease. By demonstrating that the correct assembly and trafficking of the Usher protein complex are required for ER homeostasis, the authors show a direct link from Usher mutations to ER stress and cell death. Thus, this study identifies ER stress as a new target to explore for the development of protective therapies for Usher syndrome.

Our results demonstrate that these four genes function in the development of stereocilia. Moreover, the striking similarity of the hair cell phenotypes indicates that these genes might function in the same or overlapping developmental processes, consistent with the previously proposed model that USH proteins function together in a multi-molecular complex (Kremer et al., 2006). This interpretation is further supported by our observation that all four proteins had partially overlapping subcellular distributions in hair cells (Fig. 1F–H; supplementary material Fig. S2, Fig. S3H–I′).

Because localization of these USH1 proteins in the cell bodies of hair cells has not been studied previously, we conducted several control experiments to ensure that the cell body labeling was not due to non-specific binding (supplementary material Fig. S3H) or cross-reactivity of the avidin/biotinylated enzyme detection complex (supplementary material Fig. S3I′). To substantiate further the colocalization of Ift88 and the USH proteins, we examined co-labeling of known markers of cellular organelles, Sec23, a marker of the ER-derived vesicles, and GM130, a Golgi marker (Cai et al., 2007; Yu et al., 2006; Zanetti et al., 2011). These markers did not overlap, as expected (supplementary material Fig. S4). These results thus strengthen our interpretation that the three USH1 proteins and Ift88 partially overlap in hair cell bodies.

### Cdh23 and Harmonin require Ift88 and Myo7aa for normal subcellular localization

**Fig. 2:**
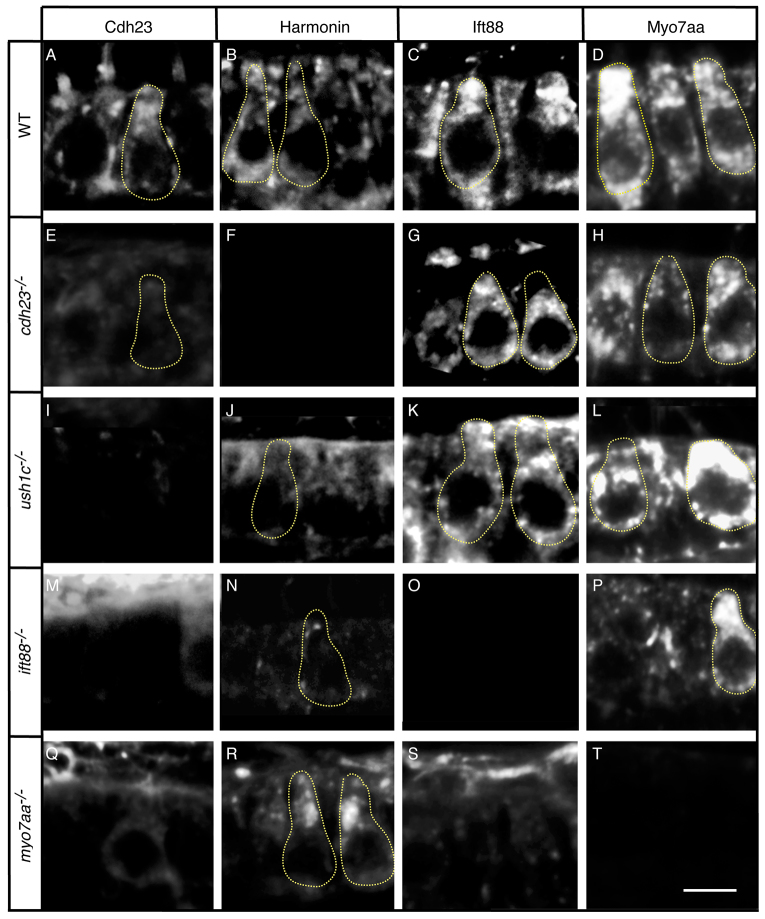
**Cdh23 and Harmonin require Ift88 and Myo7aa for normal localization in hair cells.** Immunolabeling in (A–D) wild-type siblings or (E–H) *cdh23*, (I–L) *ush1c*, (M–P) *ift88* and (Q–T) *myo7aa* mutants of (A,E,I,M,Q) Cdh23, (B,F,J,N,R) Harmonin, (C,G,K,O,S) Ift88 and (D,H,L,P,T) Myo7aa. Confocal views of anterior macula hair cells are shown. Confocal sections are 0.8 μm thick, whereas the neck and basal region of the hair cell have an estimated diameter of 2–2.5 μm and 5–6 μm, respectively. Thus, not all confocal sections contain a hair bundle, and the observed shape of the hair bundle varies according to the mounting angle (see also supplementary material Fig. S3). Some individual hair cells are outlined by dotted lines. WT, wild type. Scale bar: 5 μm.

To learn whether interactions among these proteins are required for their proper distributions in hair cells, we used protein-specific antibodies to characterize the subcellular localizations of the three USH1 proteins in wild types and in USH1 and *ift88* mutants. In wild-type animals, all four proteins were localized in the cell bodies, particularly in the subapical region (Fig. 2A–D; supplementary material Fig. S3). Ift88 and the USH1 proteins were also observed together in the kinocilium, whereas only USH1 proteins were found in the hair bundle (Fig. 2A; supplementary material Fig. S3), as previously observed in several species (Boëda et al., 2002; Grati and Kachar, 2011; Jones et al., 2008; Lagziel et al., 2005; Phillips et al., 2011; Siemens et al., 2002; Söllner et al., 2004; Tsujikawa and Malicki, 2004; Verpy et al., 2000).

Mutations in any one of the four genes resulted in a drastic reduction or an almost complete lack of Harmonin or Cdh23 immunoreactivity not only in hair bundles, but also in the hair cell bodies (Fig. 2). Ift88 and Myo7aa were less affected by mutations in *cdh23* (Fig. 2G,H) or *ush1c* (Fig. 2K,L). These results demonstrate that Cdh23, an USH1 transmembrane protein, and Harmonin, the USH1 scaffold protein, require not only the Myo7aa and Ift88 transport proteins, but also each other for proper protein levels and localization in hair cells. Lack of one of the proteins could negatively impact expression levels or stability of the other proteins. On the other hand, the persistence of Ift88 and Myo7aa proteins in *cdh23* and *ush1c* mutants argues that these proteins can be stabilized and localized subcellularly in hair cells independently of Cdh23 and Harmonin.

Previous studies showed that Cdh23 protein localizes relatively normally in stereocilia of mice lacking *Myo7a* (Boëda et al., 2002; Lefèvre et al., 2008; Senften et al., 2006). Our observation of drastically reduced Cdh23 levels in the hair cell bodies of *myo7aa* mutants seems to contradict these findings from mice. Alternatively, the difference between these studies could indicate that the homeostatic rate of protein trafficking, rather than the final distribution of proteins, is disrupted. To test this possibility, we examined localization of Cdh23 protein using ‘direct’ detection with a secondary antibody directly coupled to a fluorochrome (Alexa Fluor 488), as used in the previous mouse studies. Specificity of the antibody was assayed in *cdh23* mutants with the more sensitive AB Complex system (supplementary material Fig. S5A,C). We were unable to detect Cdh23 in the cell bodies of either wild-type or *myo7aa* mutant animals with the direct labeling method, although the antibody labeled the hair bundles (supplementary material Fig. S5B,D). This result is consistent with the previous studies in mice and further supports our conclusion that levels of Cdh23 protein are significantly reduced in hair cell bodies of *myo7aa* mutants, but Cdh23 can nevertheless reach the stereocilia, albeit in lesser amounts than in wild-type animals.

### Cdh23, Harmonin, Ift88 and Myo7aa proteins are in close enough proximity to form a complex

To examine whether the three USH1 proteins are close enough to each other to form a complex *in situ*, we adapted a proximity labeling technique (Söderberg et al., 2006) to analyze zebrafish mechanosensory hair cells. Ift88 and the USH1 proteins were recognized by specific primary antibodies, which in turn were recognized by oligonucleotide coupled secondary antibodies. When the oligonucleotides are in close proximity [estimated to be 40 nm or less (Söderberg et al., 2006)] they can guide the formation of circular DNA strands that, in turn, serve as templates for localized rolling-circle amplification that incorporates fluorochrome-labeled nucleotides. This type of assay has the specificity and sensitivity to reveal the precise subcellular localization of stable and transient protein-protein interactions *in situ* and has been used to characterize protein interactions in brain slices (Trifilieff et al., 2011), retina (Blasic et al., 2012) and photoreceptors (Wang et al., 2012).

**Fig. 3:**
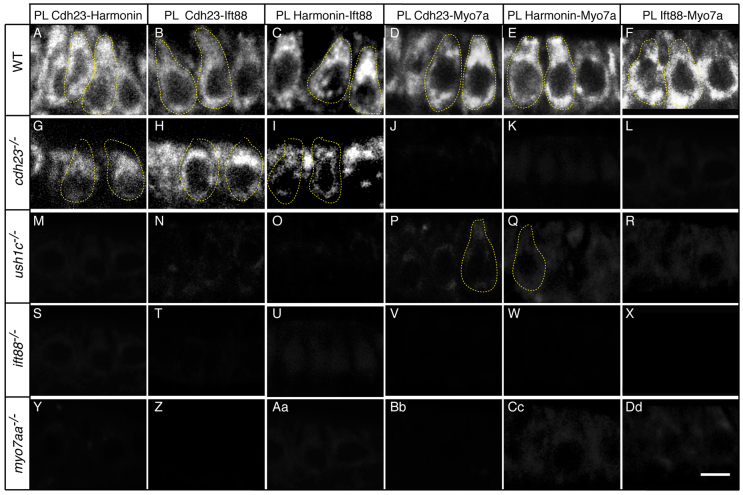
**Cdh23, Harmonin, Ift88 and Myo7aa proteins are in close proximity.** Proximity labeling (PL) of (A,G,M,S,Y) Cdh23 and Harmonin, (B,H,N,T,Z) Cdh23 and Ift88, (C,I,O,U,Aa) Harmonin and Ift88, (D,J,P,V,Bb) Cdh23 and Myo7a, (E,K,Q,W,Cc) Harmonin and Myo7a, and (F,L,R,X,Dd) Ift88 and Myo7. (A–F) Wild-type (WT) siblings; (G–L) *cdh23*, (M–R) *ush1c*, (S–X) *ift88* and (Y-Dd) *myo7aa* mutants. Confocal views of anterior macula hair cells. Some individual hair cells are outlined by dotted lines. Scale bar: 5 μm.

Analyzing proteins pairwise, we found that all four proteins are in close proximity in the cell bodies of hair cells (Fig. 3A–F), consistent with them forming a multi-molecular complex as previously proposed for the USH1 proteins based on *in vitro* binding assays (Adato et al., 2005b; Reiners et al., 2006; Siemens et al., 2002; van Wijk et al., 2006). Proximity labeling was greatly reduced or completely lacking in all four mutants (Fig. 3G,Dd), even for protein pairs where the affected gene encoded one of the other proteins. This indicated that formation of the complex was blocked by a defect in any one of its members. The one exception was in *cdh23* mutants, in which some proximity labeling signal remained for Cdh23-Harmonin (Fig. 3G), Cdh23-Ift88 (Fig. 3H) and Harmonin-Ift88 (Fig. 3I) protein pairs, but not for Myo7a (Fig. 3J,L). This result shows that an incomplete complex, lacking Myo7a, formed in this mutant. The residual signal could indicate that the available mutant allele (*cdh23^tj264a^*), which has a D166V missense mutation (supplementary material Fig. S6), does not result in complete loss of Cdh23 protein. Although Cdh23 protein was nearly undetectable in the mutant by single antibody labeling (Fig. 2E), the proximity labeling technique allowed us to detect formation of some Cdh23-Harmonin (Fig. 3G) and Cdh23-Ift88 (Fig. 3H) pairs. This interpretation was further supported by knocking down Cdh23 protein with a translation-blocking morpholino antisense oligonucleotide, which also drastically reduced the proximity labeling signals (supplementary material Fig. S7).

Because this proximity assay has not been used previously to study zebrafish hair cells, we ran a number of control experiments. To control for non-specific binding between the various secondary antibodies, we added only one or no primary antibody to the reaction and observed no signal (supplementary material Fig. S8 and data not shown). To control for non-specific binding of the primary antibodies within the cell bodies, we used the proximity assay to examine the proximity of Harmonin and acetylated tubulin or gamma tubulin (γ-tubulin). Although acetylated tubulin is distributed throughout the cytoplasm and kinocilium (supplementary material Fig. S9A–C), the proximity signal with Harmonin was confined only to the basal region of the cell body at the level of the synapses (supplementary material Fig. S9D–F), in agreement with previous reports of Harmonin localization in this region (Gregory et al., 2011; Gregory et al., 2013). Moreover, the proximity signal was absent in *ush1c* mutants (supplementary material Fig. S9G–I), further confirming its specificity. We obtained similar results with γ-tubulin (supplementary material Fig. S9J–O). Together, these results demonstrate that the proximity assay reveals specific interactions among the USH1 proteins and Ift88 in zebrafish hair cells.

**Fig. 4:**
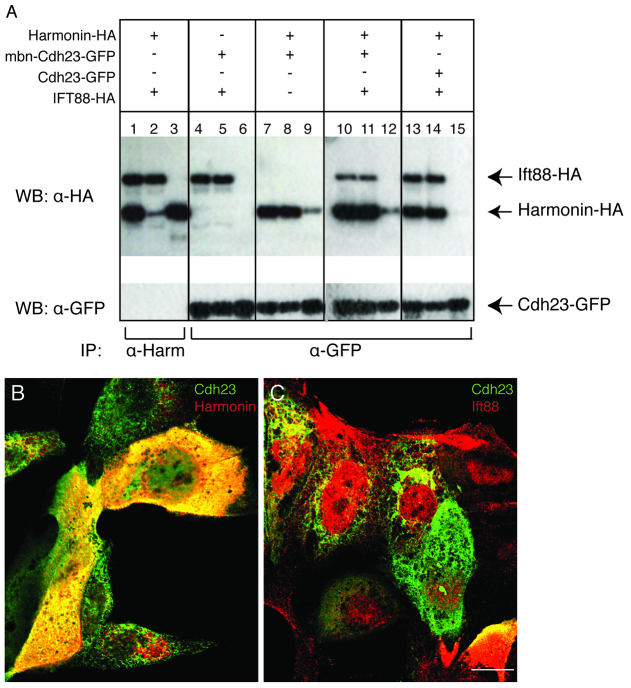
**Harmonin and Cdh23 bind directly to each other, but not to Ift88.** (A) Co-immunoprecipitation. MDCK cells were transfected as indicated by (+) with combinations of DNA vectors encoding Harmonin-HA, mbn-Cdh23-GFP, Cdh23-GFP or IFT88-HA. Input lanes: 1,4,7,10,13. Unbound fraction lanes: 2,5,8,11,14. Bound fraction lanes: 3,6,9,12,15. Immunoprecipitation was performed with an antibody against Harmonin (α-Harm) or GFP (α-GFP). (B,C) Confocal sections of transfected MDCK cells. (B) Double immunolabeling of mbn-Cdh23-GFP (green) and Harmonin (red). (C) Double immunolabeling of mbn-Cdh23-GFP (green) and Ift88 (red). Harmonin-HA: full-length zebrafish Harmonin isoform A fused to HA-tag at the C-terminal. mbn-Cdh23-GFP: zebrafish Cdh23 membrane bound cytoplasmic domain fused to GFP at the C-terminal. Cdh23-GFP: zebrafish Cdh23 cytoplasmic domain fused to GFP at the C-terminal. Ift88-HA: full-length zebrafish Ift88 fused to HA-tag at the C-terminal. Scale bar: 7.5 μm.

The positive proximity labeling signal for Ift88 with Cdh23, Harmonin and Myo7aa (Fig. 3B,C,F) was unexpected. With the exception of Spag5 (Kersten et al., 2012), previous studies have not reported direct binding between USH proteins and components of the microtubule transport system. Because the proximity assay indicates that proteins are near each other, but not necessarily binding directly, we used co-immunoprecipitation to see whether Ift88 can interact with the USH1 proteins. We expressed HA-tagged Harmonin, HA-tagged Ift88 and GFP-tagged Cdh23 in Madin-Darby canine kidney (MDCK) cells. Protein complexes were immunoprecipitated using anti-Harmonin or anti-GFP antibodies, and protein extracts were analyzed on western blots. Harmonin was able to bind to a membrane-targeted intracellular domain of Cdh23 (mbn-Cdh23; Fig. 4A, lanes 9 and 12), but neither of these USH proteins immunoprecipitated with Ift88 (Fig. 4A, lanes 3, 6, 12 and 15). Consistent with these results, immunolabeling of MDCK cells showed extensive overlap of mbn-Cdh23 with Harmonin (Fig. 4B), but little overlap with Ift88 (Fig. 4C). We obtained essentially the same results when the vectors were transfected into COS7 cells (supplementary material Fig. S10 and data not shown). Interestingly, the intracellular Cdh23 domain, without membrane tethering, did not co-precipitate with Harmonin (Fig. 4A, lane 15), consistent with the interpretation that association of Cdh23 with the membrane is required for USH complex formation. These results show that Ift88 does not co-immunoprecipitate with Cdh23 or Harmonin and suggest that Ift88 might participate in the complex with the three USH1 proteins indirectly.

### The Cdh23, Harmonin, Ift88 and Myo7aa protein complex assembles at the ER

The proximity labeling signal was very strong in the perinuclear region of hair cells (Fig. 3A–F). The ER is a major constituent of the perinuclear region, and the proximity labeling in this region suggested that the complex of Ift88 and the three USH1 proteins forms at the time of their synthesis in the ER. Membrane proteins, including Cdh23, are synthesized in the rough ER and packaged into COPII vesicles at the ER exit site (ERES). Thus, we examined the proximity of the USH proteins to Sec23 or Sec13, major components and markers of COPII vesicles (Zanetti et al., 2011).

**Fig. 5:**
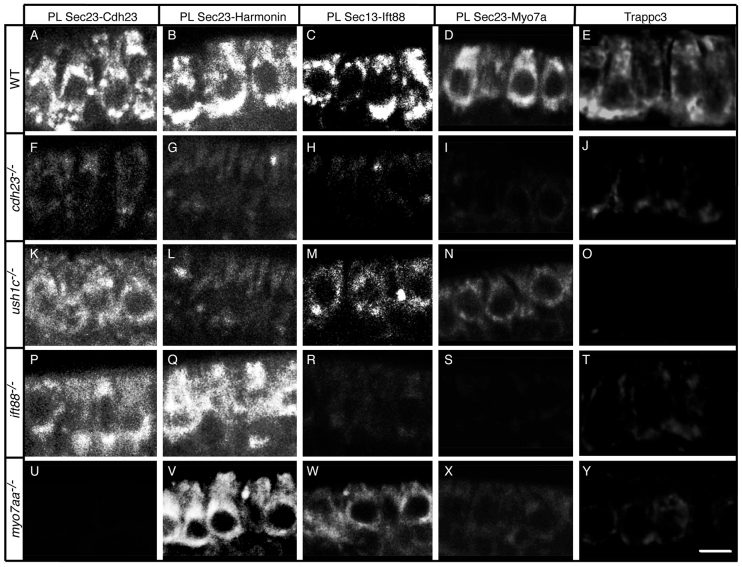
**The Cdh23, Harmonin, Ift88 and Myo7aa protein complex is present at the ER and associated vesicles.** Proximity labeling (PL) of (A–D) wild-type (WT) siblings, and (F–I) *cdh23*, (K–N) *ush1c*, (P–S) *ift88* and (U–X) *myo7aa* mutants. Proximity labeling of (A,F,K,P,U) Sec23 and Cdh23, (B,G,L,Q,V) Sec23 and Harmonin, (C,H,M,R,W) Sec13 and Ift88, and (D,I,N,S,X) Sec23 and Myo7a (recognizes Myo7aa and possibly Myo7ab; see Materials and Methods). (E,J,O,T,Y) Immunolabeling of Trappc3 in (E) WT, and (J) *cdh23*, (O) *ush1c*, (T) *ift88* and (Y) *myo7aa* mutants. Confocal views of anterior macula hair cells. Scale bar: A–D,F,I,K–N,P–S,U–X: 7.5 μm; E,J,O,T,Y: 5 μm.

Using pairwise labeling, we found that Sec23 is in close proximity to Cdh23 (Fig. 5A). Harmonin (Fig. 5B) and Myo7aa (Fig. 5D) were also in close proximity to Sec23, and Ift88 was close to Sec13 (Fig. 5C). The proximity of these proteins was also indicated by standard double labeling with antibodies specific for each protein (supplementary material Fig. S11A–C). This result was surprising, because the two USH1 cytoplasmic proteins would not normally be expected to associate with transport vesicles. Together, these results indicate that the three USH1 proteins form a complex that is packaged in COPII vesicles. The inclusion of Ift88 in this complex (Fig. 3B,C,F and Fig. 5C) indicates that COPII vesicles carrying the USH complex might be trafficked via microtubules from the ERES (Gupta et al., 2008).

In *cdh23* mutants, association of Sec23 or Sec13 with the other two USH1 proteins and Ift88 was lost (Fig. 5F–I). Presumably, misfolding of the mutant Cdh23 protein precludes association of the USH1 and Ift88 cytoplasmic proteins with COPII vesicles. Also, the Cdh23-Sec23 proximity labeling signal was completely absent in *myo7aa* mutants (Fig. 5U), indicating that interaction with Myo7aa is required for translocation of Cdh23 to the ERES. This interpretation was further supported by the apparent formation of an incomplete complex, lacking Myo7aa, in *cdh23* mutants (Fig. 3J–L). Interactions with the Sec proteins were also reduced in *ush1c* mutants (Fig. 5K–N). In *ift88* mutants, by contrast, assembly of the complex was completely disrupted (Fig. 3S–X), although Cdh23 (Fig. 5P) and Harmonin (Fig. 5Q) could still associate with Sec23, albeit at diminished levels. This shows that, in *ift88* mutants, individual Sec23 COPII vesicles contain Cdh23 or Harmonin, but not both. Together, these observations indicate that Myo7aa is required for Cdh23 translocation to the ERES, where Ift88-dependent stabilization and secretion of the USH1 protein complex normally occurs. The binding of Harmonin to the membrane-bound form of Cdh23 (Fig. 4A, lanes 9 and 12) but not the cytoplasmic form (Fig. 4A, lane 15) provides additional support that Cdh23 needs to be transported through the ERES for assembly of the complex.

**Fig. 6:**
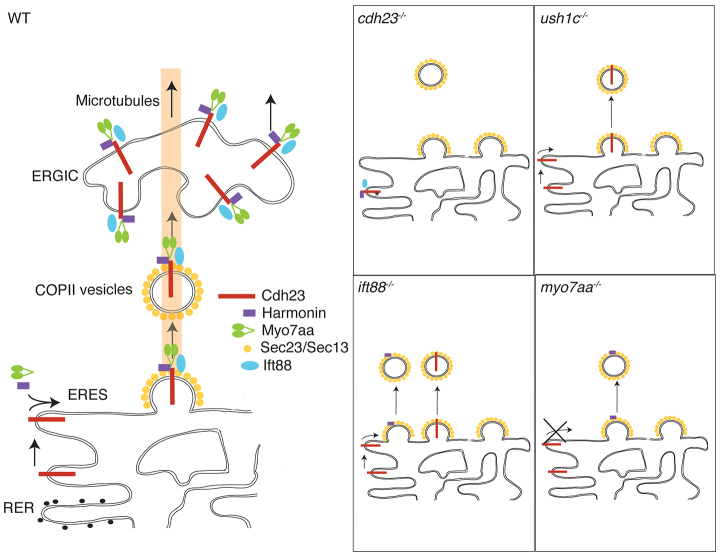
**The Cdh23, Harmonin, Ift88 and Myo7aa protein complex preassembles at the ER.** Model for assembly and trafficking of USH proteins. Under normal conditions in wild-type (WT) animals, Cadherin23, Harmonin, Ift88 and Myo7aa preassemble into a complex at the level of the ER. Interaction between Cdh23 and Myo7aa is required for Cdh23 translocation to the ERES. All four proteins are required for assembly of a functional complex. Once assembled, the complex translocates to ER exit sites (ERES), engages components of the COPII system (Sec23 and Sec13), and enters the secretory pathway where it is trafficked to the ER-Golgi intermediate complex (ERGIC) en route to final destinations. Loss-of-function mutations in *cdh23*, *ush1c*, *ift88* and *myo7aa* disrupt assembly of the complex in various ways. RER, rough endoplasmic reticulum.

The loss of Sec23 or Sec13 association with the three USH1 proteins or Ift88 in USH1 or *ift88* mutants indicated that assembly of the complex is necessary for targeting the complex to the ERES and subsequent secretion. To test this interpretation further, we examined formation of the ER-Golgi intermediate complex (ERGIC), as indicated by Trappc3 labeling (Cai et al., 2007; Yu et al., 2006). In wild-type hair cells, some Trappc3 labeling overlapped with that of each of the three USH1 proteins (supplementary material Fig. S11D–F), indicating that the USH1 proteins traffic through the ERGIC. The total amount of Trappc3 labeling was reduced in all three USH1 mutants and in *ift88* mutants (Fig. 5E,J,O,T,Y; supplementary material Fig. S12). This result is consistent with the trafficking defects seen by proximity labeling with Sec23 or Sec13 (Fig. 5), and further demonstrates that defects in assembly or trafficking of the complex produce a global reduction of the ERGIC. Thus, defects in Ift88 or any one of the three USH1 proteins disrupt formation of the complex, trafficking, and biogenesis of the ERGIC (Fig. 6).

### The Cdh23, Harmonin, Myo7aa and Ift88 protein complex is required for trafficking of USH2 proteins and can trigger ER stress when defective

**Fig. 7:**
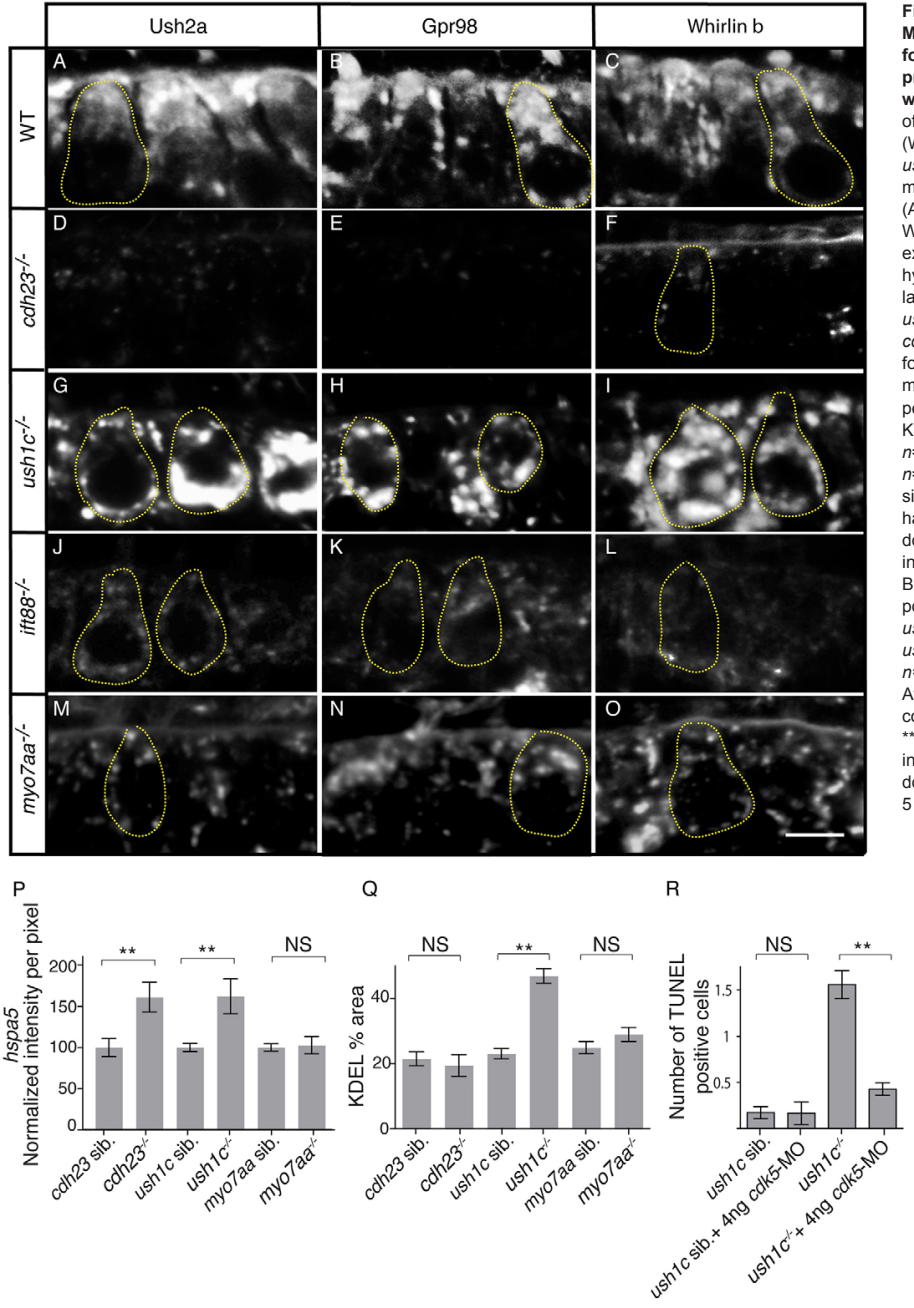
**The Cdh23, Harmonin and Myo7aa protein complex is required for proper trafficking of USH2 proteins and can trigger ER stress when defective.** (A–O) Immunolabeling of USH2 proteins in (A–C) wild-type (WT) siblings, and (D–F) *cdh23*, (G–I) *ush1c*, (J–L) *ift88* and (M–O) *myo7aa* mutants with antibodies against Ush2a (A,D,G,J,M), Gpr98 (B,E,H,K,N) and Whirlinb (C,F,I,L,O). (P) Levels of *hspa5* expression based on *in situ* hybridization technique in whole-mount larvae. For *ush1c* siblings *n*=57; *ush1c^−/−^* *n*=51; *cdh23* siblings *n*=35; for *cdh23^−/−^* *n*=33; *myo7aa* siblings *n*=24; for *myo7aa*^−/−^ *n*=24 analyzed anterior maculae. (Q) Quantification of percentage area per hair cell containing KDEL expression. For *ush1c* siblings *n*=85; *ush1c^−/−^* *n*=125; *cdh23* siblings *n*=66; for *cdh23^−/−^* *n*=60; *myo7aa* siblings *n*=60; *myo7aa*^−/−^ *n*=54 analyzed hair cells for each genotype. (R) Knockdown of *cdk5* rescues ER-stress-induced cell death in the *ush1c* mutant. Bars show average number of TUNEL-positive cells. For *ush1c* siblings *n*=52; *ush1c* siblings + 4 ng *cdk5-*MO *n*=18; *ush1c^−/−^* *n*=70; *ush1c^−/−^* + 4 ng *cdk5-*MO *n=*70 analyzed anterior maculae. Average ± s.d. Statistics were conducted with Student’s *t*-test. **P<0.01. NS, non significant. Some individual hair cells are outlined by dotted lines. MO, morpholino. Scale bar: 5 μm.

Given the importance of close association of Cdh23, Harmonin and Myo7aa for assembly and intracellular trafficking of this complex of USH1 proteins and the requirement of Myo7a for the subcellular localization of Gpr98, whirlin and usherin in mouse mechanoreceptors (Michalski et al., 2007), we also examined the cell body localization of the USH2 proteins Ush2a, Gpr98 and Whirlinb. In addition to their localization in hair bundles (supplementary material Fig. S13), consistent with mouse and human (Adato et al., 2005a; Bonnet and El-Amraoui, 2012; Mburu et al., 2003; Michalski et al., 2007), all three proteins were localized subapically in wild-type mechanosensory hair cells (Fig. 7A–C). In *cdh23* (Fig. 7D–F), *ift88* (Fig. 7J–L) and *myo7aa* (Fig. 7M–O) mutants, all three USH2 proteins were greatly reduced in the cell body. In *ush1c* mutants (Fig. 7G–I), the USH2 proteins were concentrated in the perinuclear region instead of subapically (supplementary material Fig. S13G–I, Fig. S14F–H). These results indicate that trafficking of the USH2 proteins from the ER was disrupted.

Problems with protein targeting to and secretion from the ERES can lead to ER stress (McGuckin et al., 2010). Because our analysis of USH1 mutants indicated defects in trafficking from the ER, we examined markers of ER stress, including *hspa5* expression (Kozutsumi et al., 1988; Kroeger et al., 2012), and the size of the ER as indicated by the area of the cell occupied by proteins that contain the signal peptide KDEL (Munro and Pelham, 1987). Expression of *hspa5* was significantly increased in *ush1c* and *cdh23* mutants (Fig. 7P), consistent with increased ER stress. In *myo7aa* mutants, however, *hspa5* expression was relatively unaffected, which might indicate partial compensation by the *myo7ab* duplicate gene. Similarly, the amount of ER membrane was expanded in *ush1c* mutants as indicated by increased KDEL labeling (Fig. 7Q; supplementary material Fig. S14E) and increased perinuclear localization of USH2 proteins (Fig. 7G–I; supplementary material Fig. S14F–H), which is further indication that the hair cells were undergoing ER stress in this mutant. Interestingly, although the amount of ER membrane apparently increased in *ush1c* mutants (Fig. 7Q), the amount of ERGIC decreased (Fig. 5E,O). Thus, the flow of membrane from the ER through the ERGIC might be compromised in USH mutants, and this could contribute to the reduced numbers of stereocilia we observed in mutant hair cells (Fig. 1). ER stress also attenuates translation (McGuckin et al., 2010), perhaps accounting for the decreased levels of USH proteins we observed (Fig. 2).

Chronic ER stress can lead to apoptosis mediated by the Cdk5-Mekk1-JNK pathway (Kang et al., 2012). Because apoptosis is a major symptom of Usher syndrome, we examined whether there is a link between ER stress and cell death in USH mutants. We used TUNEL labeling to assay apoptosis of hair cells. Consistent with our previous study (Phillips et al., 2011), we observed increased levels of cell death in *ush1c* mutants relative to wild-type siblings (Fig. 7R). Knock-down of Cdk5 activity by morpholino injection blocked the increased cell death in the mutants, demonstrating that ER stress in these cells leads to apoptosis.

## DISCUSSION

Our studies show that Cdh23, Harmonin, Myo7aa and Ift88 are in close enough proximity to form a multi-molecular protein complex, and this interaction occurs at the level of the ER in zebrafish inner ear mechanosensory hair cells. Assembly of this complex is required for trafficking of USH2 proteins and for biogenesis of the ERGIC. Defects in complex formation lead to ER stress and apoptosis. Together, our results demonstrate that normal formation and trafficking of this complex are required for proper function of the common secretory pathway and subsequent development of hair bundles.

The use of an enhancement step during antibody detection allowed us to study variations in protein levels in the cytoplasm, as well as hair bundles, in various genetic backgrounds. The localization of the USH proteins at the level of the hair bundle in zebrafish at 5 days post-fertilization (dpf) corresponds to that in mouse during embryonic stages, just before the spatial restriction of the USH proteins along the stereocilia occurs (Adato et al., 2005a; Boëda et al., 2002; Lagziel et al., 2005). In early postnatal stages, whirlin has been localized near both the tip and ankle links (Grati et al., 2012), and also along the stereocilia (Mburu et al., 2003). Gpr98 has been studied primarily after birth, so its distribution during early development is less well known in mice.

Assembly of the Cdh23, Harmonin, Myo7a and Ift88 complex could couple membrane targeting to the actin polymerization and stabilization machinery required for stereocilia growth. The initial localization of Cdh23, Harmonin and Myo7a at the kinocilium (Boëda et al., 2002; Lagziel et al., 2005; Lefèvre et al., 2008) indicates that USH1 proteins engage in ciliary trafficking. Ift88 might facilitate the movement of proteins like Cdh23, Harmonin and Myo7a across the ciliary barrier (Nachury et al., 2010) towards the apical region during formation of stereocilia (Tanimoto et al., 2011; Tilney et al., 1992). Consistent with this idea, Cdh23 has been reported at the level of the centrosome (Lagziel et al., 2005), a structure that could be used as a cargo loading point for vesicular ciliary trafficking.

Our results show that, although assembly of the USH1 protein complex and subsequent trafficking are seriously disrupted in USH1 mutants, some USH1 protein eventually localizes properly in the developing stereocilia (supplementary material Fig. S5D). Thus, failure of USH1 protein complex assembly does not cause ‘all or none’ effects, but rather differentially alters protein distributions. Similarly, mutant *Myo7a*, *Ush1c* and *Cdh23* mice form fewer mechanoreceptor lateral links (Lefèvre et al., 2008), consistent with decreased trafficking of these components. It is still unclear, however, precisely how failure of assembly of this USH1 complex affects protein trafficking routes in the hair cell.

At least three hypotheses could explain the underlying trafficking mechanism affected by disruption of the USH1 complex. First, the complex could function as a cargo adaptor. Although SEC23A, the core element of the secretory pathway, is ubiquitously expressed, human patients and zebrafish with *SEC23A* mutations have tissue-and cell-specific phenotypes with no reported mechanosensory dysfunction (Fromme et al., 2007; Lang et al., 2006) (and data not shown). This suggests that, in *SEC23A*-deficient hair cells, USH proteins are trafficked correctly and that the trafficking phenotype we have observed is specific to USH mutants. Previous authors have proposed that the trafficking efficiency of the secretory pathway is modulated in a cell-type- and tissue-specific manner by specific interactions between cargo and core elements of the secretory pathway (Fromme et al., 2007). Our discovery of the required function of USH proteins in trafficking thus suggests that the assembled protein complex of Cdh23, Harmonin, Myo7aa and Ift88, rather than the individual components, functions as a new cargo adaptor that sorts transmembrane proteins among membrane transport carriers.

Second, the Cdh23, Harmonin, Myo7aa and Ift88 complex might function as a cargo effector required for vesicle transport. It has been previously shown that the vesicular recruitment and motor activity of Myo5a, an atypical myosin (like Myo7aa), depends on the function of distinct Rab GTPases (Wu et al., 2001; Wu et al., 2002). We found that the USH complex can interact with Sec23, and that disruption of complex assembly leads to defects in the biogenesis of the ERGIC, as assayed by Trappc3 labeling. Previous studies showed that Trappc3 interacts physically with Sec23, and that this interaction is required for homotypic fusion of COPII vesicles (Cai et al., 2007; Yu et al., 2006). Trappc3 is an essential subunit of the TRAPP I complex, which acts as a guanine exchange factor for Rab1 (Cai et al., 2007; Jones et al., 2000; Kim et al., 2006; Wang et al., 2000). Thus, in hair cells, the Cdh23, Harmonin, Myo7aa and Ift88 complex might play a structural role during biogenesis of the ERGIC by providing a physical platform for the required interaction between Trappc3 and Sec23 during homotypic fusion of COPII vesicles.

A third hypothesis is that the observed trafficking defects are secondary consequences of ER stress and induction of the unfolded-protein response (UPR) pathway. The combination of both responses could induce trafficking failure. Structural integrity of the ERGIC has been used as a read-out of functional trafficking through the secretory pathway. Disrupted cycling of the cargo receptors Surf4, ERGIC53, p25 and Syntaxin17 affects biogenesis of the ERGIC (Mitrovic et al., 2008; Muppirala et al., 2011) owing to disequilibrium of membrane flow among secretory-pathway compartments. Contrary to this hypothesis, our observation that *myo7aa* and *ift88* mutants do not develop elevated *hspa5* levels or ER distension, at least by 5 dpf (Fig. 7), indicates that trafficking defects do not necessarily cause ER stress. Thus, further study is required to provide an understanding of the underlying mechanisms that will ultimately explain how disruption of the USH1 protein complex affects membrane flow from the ER.

The rough ER is a major site of secreted and membrane-integrated protein synthesis, including, presumably, the USH transmembrane proteins. Post-translational modification and folding of proteins occurs in the ER. Protein folding is regulated by weak, primarily hydrophobic, interactions and, thus, a proportion of all proteins normally misfold. Because of the likelihood of misfolding, the ER has mechanisms that are part of routine housekeeping to recognize misfolded proteins and remove them for degradation (Vembar and Brodsky, 2008). A range of molecules within the ER, including chaperones, are essential for appropriate biosynthesis and correct folding. Chaperones disengage from proteins once the correct conformation is achieved, and, conversely, chaperones accumulate when proteins misfold. If protein misfolding is excessive, ER homeostasis is not maintained, leading to ER stress. Chaperone production is also upregulated in response to increased misfolding, and measurement of increased chaperone production, particularly HSPA5 (also called BIP or GRP78), is commonly used as a marker of ER stress (Kozutsumi et al., 1988; Kroeger et al., 2012). ER stress is often transient and well controlled; however, prolonged ER stress can seriously affect protein production and other cellular functions, ultimately triggering apoptosis (Doyle et al., 2011).

Our observations of defects in protein trafficking, protein retention in the ER, upregulation of *hspa5* chaperone transcription and increased size of the ER in USH1 mutants indicate that these mutations lead to ER stress. Our studies also show that some USH proteins preassemble into a complex at the ER and that defects in one or more components of the complex can activate the UPR. Perhaps when one member of the complex is defective or missing, surfaces on other members of the complex are abnormally exposed and might be recognized as ‘misfolded’, thus triggering the UPR. With prolonged absence or misfolding of one component of the USH complex, the UPR system could be overwhelmed, leading to ER stress. ER stress also activates the Cdk5-Mekk1-JNK cell-death pathway (Kang et al., 2012). Thus, in USH, a long-term consequence of USH gene mutations is ER stress that ultimately leads to apoptosis.

Recent studies of neurodegenerative diseases (Doyle et al., 2011) such as Alzheimer’s, Parkinson’s and some retinopathies (Kang et al., 2012; Kroeger et al., 2012; Kunte et al., 2012; Shinde et al., 2012) have implicated ER stress as a probable cause of apoptosis. Our current and previous studies (Ebermann et al., 2010; Phillips et al., 2011) add to this view by demonstrating both ER stress and apoptosis in zebrafish USH gene mutants, thus providing the first evidence that ER stress is a proximal cause of sensory cell loss in USH. The link between ER stress and apoptosis raises the possibility that therapeutics that are being developed for the treatment of other neurodegenerative diseases (Doyle et al., 2011) will be helpful in managing the progression of symptoms in USH patients. Although hearing defects are typically congenital in USH1 owing to defects in the mechanoreceptors, hair cells ultimately die in USH1 mutants. In all forms of USH, vision loss is progressive and photoreceptors degenerate over decades. Treatments that delay or reduce cell loss will provide time to patients, while therapies that address the defects can be developed and applied to the remaining, non-degenerating cell populations.

## MATERIALS AND METHODS

### Animals

Zebrafish strains were AB wild-type, *cdh23^tj264a^* (Söllner et al., 2004), *ush1c^fh293^* (Phillips et al., 2011), *ift88^tz288b^* (Tsujikawa and Malicki, 2004) and *myo7aa^ty220d^* (Ernest et al., 2000). All mutations are recessive alleles; we refer to animals with homozygous mutant alleles as mutants. Phenotypically wild-type siblings were used as controls. Animals were raised in a 10-hour dark and 14-hour light cycle and maintained as previously described (Westerfield, 2007). Larvae were staged according to the standard series (Kimmel et al., 1995). Results presented were obtained at 5 dpf. All animal-use protocols were IACUC approved.

### Constructs

To construct Ush1c-HA and Ift88-HA, the coding sequence of the HA-tag was fused to the 3′ ends of *ush1c* (Phillips et al., 2011) and *ift88* (cloned using the following PCR primers, forward: 5′-ATGGAGAATGTGCATC -TTGTC-3′; and reverse: 5′-CTCTGATCAAATATAGTTCAGCATATT-3′), respectively. For mbn-Cdh23-GFP, the sequence encoding the signal peptide and membrane-targeting domain of *syncam2* (Doyle et al., 2011) was fused to the 5′ end of the sequence that encodes the intracellular domain of *cdh23*, which includes exon 68. The GFP coding sequence was then fused to the 3′ end of the construct. For Cdh23-GFP, only the GFP sequence was fused to the *cdh23* 3′ end. The *hspa5* clone was obtained from the ZIRC EST bank (cb865) and then used as a template to make a full-length antisense RNA probe for *in situ* hybridization. All gene and protein names are approved ZFIN (http://zfin.org) nomenclature.

### Whole-mount immunohistochemistry and proximity ligation assay

Larvae were anesthetized and fixed at 5 dpf in BT fix (4% PFA, 4% sucrose, 0.15 mM CaCl_2_ in PBS) for 48 hours for immunolabeling or 24 hours for the proximity ligation assay, washed in PBST (PBS + 0.01% Tween20), and processed for immunolabeling. Samples were permeabilized in 5% Tween20 in PBS for 9 hours, fixed for 20 minutes in BT-fix, then blocked for at least 2 hours in PBS blocking solution (PBS + 0.01% Tween20, 10% NGS, 10% BSA), followed by an overnight incubation at 4°C in primary antibody solution (PBS blocking + primary antibody). For double immunolabeling, both primary antibodies were added simultaneously. Unbound antibodies were removed by five serial incubations of 30 minutes in PBST. Samples were fixed for 20 minutes in BT-fix, re-incubated in PBST for short periods, blocked, and incubated overnight at 4°C in secondary antibody solution (PBS blocking + secondary antibody). Unbound secondary antibodies were removed as described above.

To detect signals, reagents A and B (AB complex) from the Vectastain ABC Elite kit (Vector Laboratories) were diluted at 1:100 each in blocking solution (AB solution) and pre-incubated for 20 minutes. Samples were then incubated for 25 minutes in AB solution, followed by at least four incubations of 5 minutes in PBST. Tyramide from the TSA kit (Perkin-Elmer) was diluted 1:50 in pre-warmed buffer reagent and added to samples for 20 minutes following the manufacturer’s recommendations. The detection reaction was stopped by adding cold TNT (0.1 M Tris-HCl, pH 7.5, 0.15 M NaCl, 0.1% Tween20) to the samples, followed by several TNT washes. For single labeling, the last TNT wash was replaced by Vectashield solution (Vector Labs). For double labeling, samples were fixed in TNT-fix (1% PFA, 0.15 mM CaCl_2_, 4% sucrose in TNT), washed in TNT, and then incubated in pre-warmed buffer reagent to quench HRP. Samples were then incubated sequentially for 1 hour with reagent A followed by reagent B of the avidin/biotin blocking kit (Invitrogen). After blocking with TNT Block (TNT + 10% NGS, 10% BSA) for 2 hours, samples were incubated overnight at 4°C in TNT secondary antibody solution (secondary antibody diluted 1:250 in TNT Block). Signal was detected as described above. After detection, samples were immersed in Vectashield reagent (Vector Labs).

For the proximity ligation assay (Duo-Link), larvae were prepared as above and treated according to the manufacturer’s recommendations (Olink Bioscience) with some minor modifications. Primary and PLA probe-coupled antibodies were diluted in PBS blocking solution, and samples were incubated overnight at 4°C. Unbound antibodies were removed through five serial PBST rinses of 30 minutes each. Samples were incubated in PBST at 37°C for 1 hour. They were then incubated at 37°C for 4 hours in Duo-Link ligation solution, followed by overnight incubation at 37°C with Duo-Link amplification solution. Samples were washed in PBST and then immersed in Vectashield for imaging.

Primary antibodies used were rabbit anti-Trappc3 (Sigma); goat, rabbit and chicken anti-Cdh23 (Santa Cruz, Sigma, and custom made against C-terminal region, respectively); rabbit anti-Cip98a (Whirlinb; SDIX); rabbit anti-GFP (Torrey Pines Biolabs); mouse anti-Gm130 (BD Transduction Laboratories); rabbit anti-Gpr98 (SDIX); goat, rabbit and guinea pig anti-Harmonin (SDIX and custom made against the C-terminal region, respectively); rabbit anti-Ift88 (Krock and Perkins, 2008); mouse anti-KDEL (Calbiochem); guinea pig anti-Myo7aa (custom made against C-terminal region); rabbit anti-Sec23 (Santa Cruz); goat anti-Sec13 (Santa Cruz); mouse anti-acetylated tubulin, used at 1:100 (Santa Cruz); mouse anti-γ-tubulin, used at 1:100 (Sigma); and rabbit anti-Ush2a (SDIX). We also used mouse anti-Myo7a (Sigma), which we showed cross-reacts with zebrafish Myo7aa (supplementary material Fig. S15). Given the sequence similarity between zebrafish Myo7aa and Myo7ab, it is possible that the antibody also recognizes Myo7ab. Secondary antibodies used were biotinylated anti-mouse, biotinylated anti-goat, biotinylated anti-rabbit, biotinylated anti-chicken and biotinylated anti-guinea pig (Vector Labs).

For whole-mount antibody labeling with the AB complex, antibodies were diluted at 1:500; secondary antibodies were used at a dilution of 1:250. For direct detection, primary anti-Cdh23 antibody was diluted at 1:250; secondary antibody coupled to a fluorochrome (Molecular Probes) was diluted at 1:250. For antibody dilutions used in cell culture immunolabeling, see ‘Cell culture and immunolabeling’ section below. For the proximity ligation assay, primary antibodies were co-incubated at 1:500, except for the anti-acetylated tubulin and γ-tubulin that were used at 1:100. The proximity ligation assay probe-coupled antibodies used were anti-goat plus, anti-goat minus, anti-rabbit plus, anti-rabbit minus, anti-mouse plus and anti-mouse minus. All proximity ligation assay probe-coupled antibodies were diluted following the manufacturer’s instructions (1:5). For immunolabeling of F-actin, FITC-phalloidin was used as described previously (Haddon and Lewis, 1996).

### TUNEL labeling

Apoptag plus Peroxidase *In Situ* Apoptosis Detection Kit was used to examine cell death following the manufacturer’s instructions with minor modifications. Larvae were fixed and permeabilized as described (Thisse and Thisse, 2008). After proteinase K treatment, larvae were incubated in acetone and ethanol solution (1:2 mixture) for 8 minutes at −20°C, followed by PBS washes. Larvae were equilibrated for 1 hour at room temperature in equilibration buffer, then incubated in reaction solution (16 μl of TdT enzyme + 38 μl of reaction buffer) for 90 minutes at 37°C. The reaction was stopped by adding 200 μl of stop buffer. The reaction product was detected with an anti-DIG coupled to HRP with DAB as substrate.

### Cell culture and immunolabeling

MDCK cells or COS7 cells were cultured in Dulbecco’s modified Eagle’s medium supplemented with 10% fetal bovine serum and penicillin (40 U/ml)/streptomycin (40 μg/ml) at 37°C under 5% CO_2_. Cells were transiently transfected with 1 μg of plasmids of interest using Lipofectamine 2000.

For cell-culture labeling, cells were cultured on poly-L-lysine-coated glass coverslips and transfected. 24 hours after transfection, cells were fixed with 4% PFA in 4% sucrose-PBS for 10 minutes and then permeabilized with 0.25% Triton X-100 in PBS. Detection was performed using mouse anti-GFP (Abcam, 1:500), rabbit anti-Harmonin (SDIX, 1:100) and rabbit anti-Ift88 (Krock and Perkins, 2008) antibodies. Secondary antibodies were goat anti-mouse Alexa-Fluor-488-conjugated and goat anti-rabbit Alexa-Fluor-568-conjugated (Molecular Probes, 1:250).

### Co-immunoprecipitation and western blotting

At 16 hours after transfection (see above), cells were lysed in 125 μl lysis buffer (150 mM NaCl, 50 mM Tris-HCl, 0.5 mM EDTA, 0.1% Triton, and protease inhibitor tablets) for 20 minutes at 4°C with agitation. The mixture was centrifuged at 5000 rpm for 5 minutes, and the supernatant was collected. 40 μl of the supernatant was kept for input, and the rest was incubated overnight at 4°C with either rabbit anti-GFP (Torrey Pines Biolabs) or rabbit anti-Harmonin (SDIX) antibodies at a concentration of 1 ng/μl. Proteins were precipitated with protein A-sepharose beads, washed three times in lysis buffer, and eluted with sample buffer. The eluted proteins were boiled, separated by SDS-polyacrylamide gel electrophoresis, and transferred to PVDF membranes. Detection was performed using rabbit anti-GFP (1:500, Torrey Pines Biolabs) or mouse anti-HA (1:500, Covance) antibodies. Secondary antibodies were donkey anti-rabbit and donkey anti-mouse coupled to HRP (1:5000, Jackson ImmunoResearch Laboratories).

### Morpholino injection

We used a Narishige microinjector to inject one-cell-stage zebrafish embryos with 2 ng of translation blocker *cdh23*_morpholino (Söllner et al., 2004) or 4 ng of the translation blocker *cdk5*_morpholino (5′-CCAGCTTCTCAT -ACTTTTGCATGGT-3′) (Easley-Neal et al., 2013). Efficiency of the *cdk5*_morpholino was evaluated as previously described (Tanaka et al., 2012). Injection volumes were estimated using a micrometer.

### Fluorescence *in situ* hybridization

Whole-mount *in situ* hybridization was carried out as previously described (Thisse and Thisse, 2008) with the following modifications: Digoxigenin-labeled probes were prepared according to the manufacturer’s instructions (Roche). Probe signal was detected using an anti-DIG POD conjugated antibody and TSA (Perkin-Elmer).

### Imaging

Vectashield immersed larvae were mounted laterally in a slide chamber and imaged with Zeiss LSM5 or Bio-Rad Radiance 2100 confocal microscopes using a 63× objective. For whole-mount and cell-culture labeling, optical sections were 0.8 μm thick. Optical sections for proximity ligation experiments were 2.5 μm thick. Images were assembled using Adobe Photoshop and Illustrator. ImageJ (v 1.43u) was used to measure *hspa5* pixel intensity and to calculate the size of KDEL-positive cell areas.

## Supplementary Material

Supplementary Material
