# Supplementary material for: Complexes of Usher proteins preassemble at the endoplasmic reticulum and are required for trafficking and ER homeostasis
Source: Dis Model Mech. 2014 Mar 13;7(5):547–59. doi: 10.1242/dmm.014068 (PMC4007406; doi:10.1242/dmm.014068)
Supplement: Supplementary Material [file supp_7_5_547__index.html]

Complexes of Usher proteins preassemble at the endoplasmic reticulum and are required for trafficking and ER homeostasis — Supplementary Material 

# Complexes of Usher proteins preassemble at the endoplasmic reticulum and are required for trafficking and ER homeostasis

## DMM014068 Supplementary Material

**Files in this Data Supplement:**

- **Supplementary Material**
