# Supplementary material for: Complexes of Usher proteins preassemble at the endoplasmic reticulum and are required for trafficking and ER homeostasis
Source: Dis Model Mech. 2014 Mar 13;7(5):547–59. doi: 10.1242/dmm.014068 (PMC4007406; doi:10.1242/dmm.014068)
Supplement: Supplementary Material [file supp_7.5.547_DMM014068.pdf]

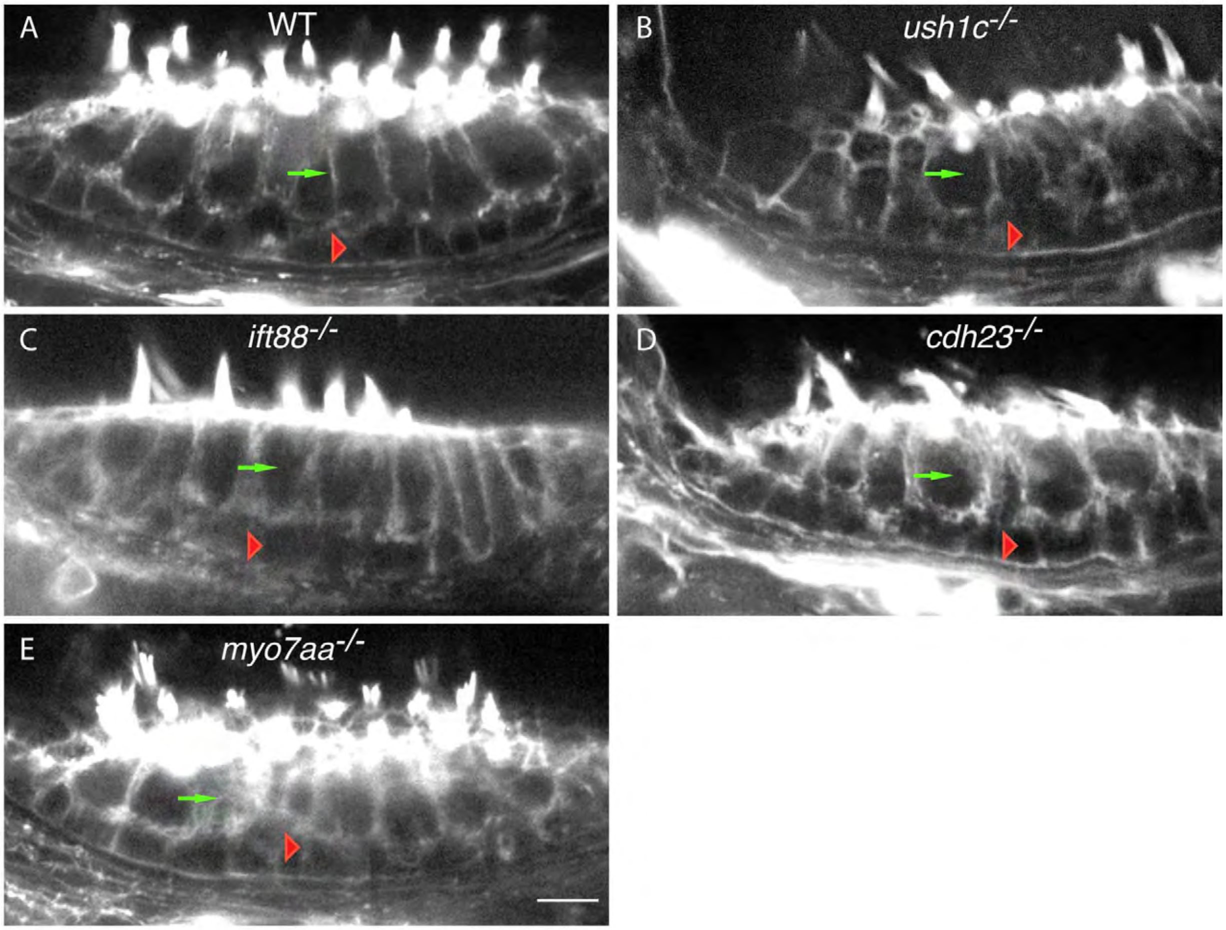

Fig. S1. Epithelial organization of the anterior macula is unaffected in USH1 and Ift88 mutants. Phalloidin labeling of anterior maculae in **(**A) wild-type, (B) *ush1c*, (C) *ift88*, (D) *cdh23*, and **(**E) *myo7aa* animals. Green arrows indicate hair cells; red arrowheads indicate supporting cells. WT: wild-type. Scale bar: 5 μm.

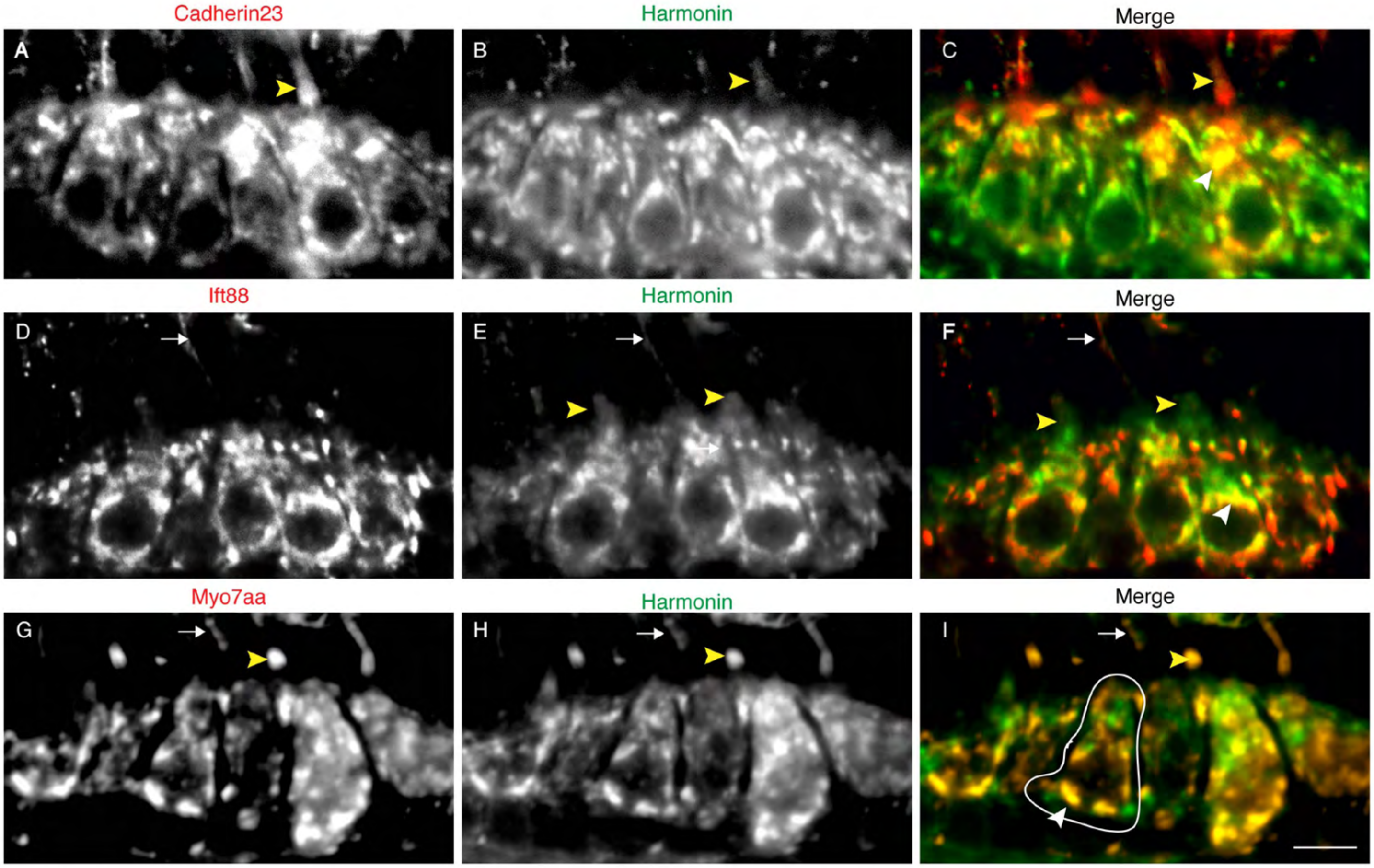

Fig. S2. Colocalization of USH1 and Ift88 proteins in the hair cell. Double immunolabeling of Cdh23 (A), Ift88 (D), Myo7aa (G), and Harmonin (B,E,H). Panels C,F, I show merged panels of A and B, D and E, G and H, respectively. Fig. 1F,G are insets of panels C and F. White arrowheads: cell body colocalization. Yellow Arrowheads: hair bundle localization. Arrows indicate kinociliary labeling. A cell body is highlighted in white. Scale bar: 5μm.

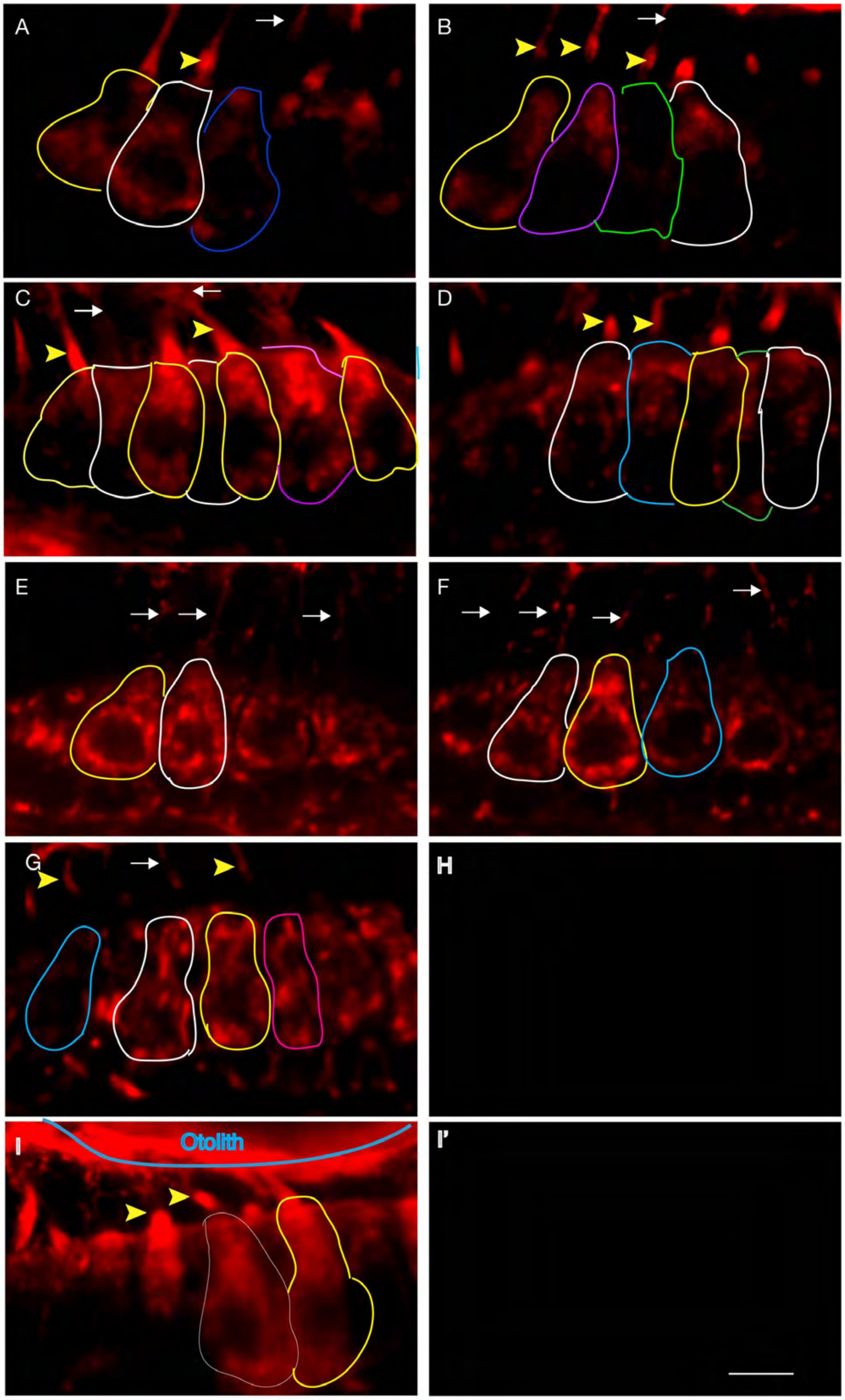

Fig. S3. Cdh23, Harmonin, Myo7aa and Ift88 localize at the level of the mechanoreceptor. (A-I´) Wild-type zebrafish anterior maculae immunolabeled for Cdh23 (A,B), Harmonin (C,D,I), or Ift88 (E,F). As a control for the binding specificity of the AB Complex, primary antibody was omitted during the reaction (H). As a control for the specificity of the second antibody detection reaction, the first reaction was developed (I), then the AB complex was quenched and blocked (see material and methods), followed by omission of the other primary antibody during the reaction (I´). Both controls resulted in an absence of signal. Confocal sections focused on the hair bundle region. Note the appearance of the hair bundle depends on its spatial distribution along the anterior macula and on the mounting angle during image acquisition (compare panels A-G,I). Yellow arrowheads: hair bundle localization. Arrows: kinociliary localization. Cell bodies in different plans are highlighted in different colors. Scale bar: 5 μm

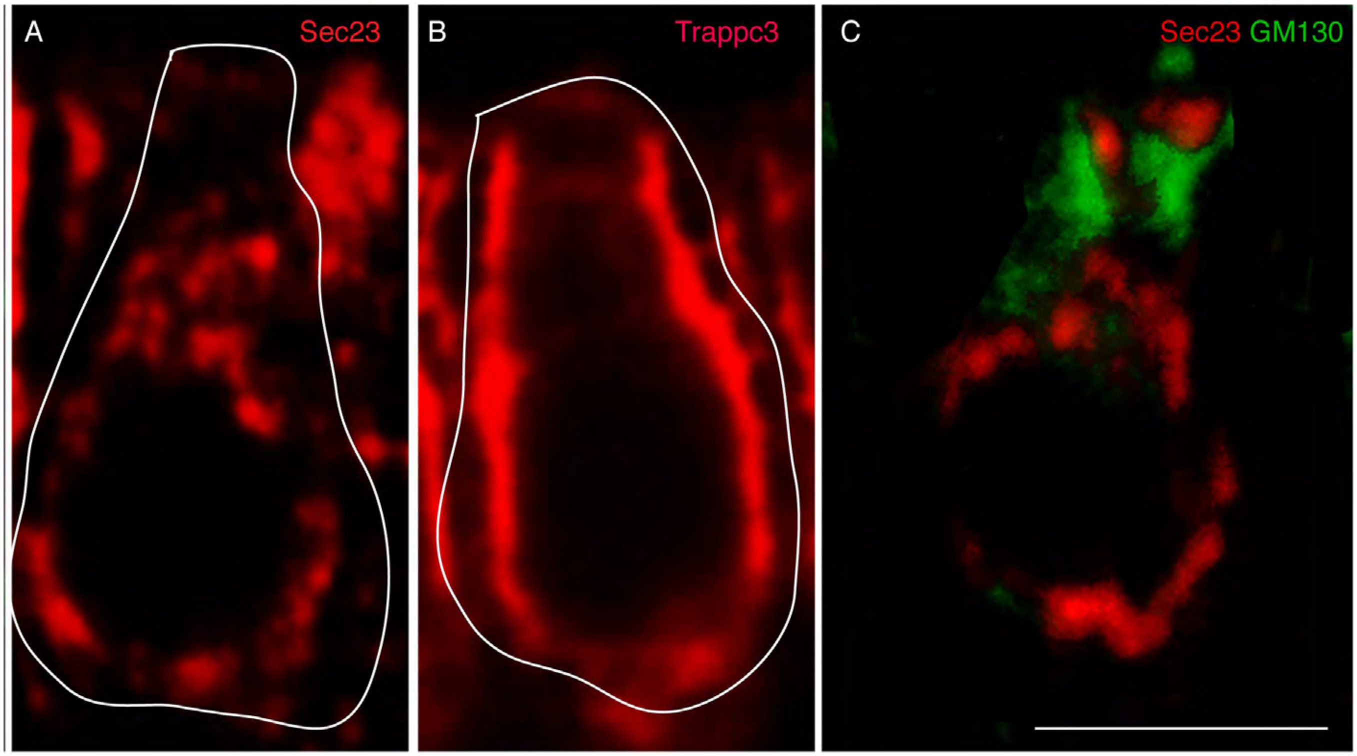

Fig. S4. AB Complex detection method resolves cytoplasmic protein pools. Proteins that participate in the secretory pathway leave the ER via budding vesicles labeled by Sec23, a component of the COPII machinery (A). Trappc3 mediates fusion of Sec23 vesicles that form the ER-Golgi intermediate compartment (ERGIC) (B). After fusion, Sec23 and other COPII components return to the ER. Vesicles budded from the ERGIC then fuse with the membranes of the Golgi apparatus, labeled by GM130 (C). GM130 and Sec23 do not co-localize despite both having broad cytoplasmic labeling (C). This indicates that colocalization signals detected with the AB complex method are specific and not due to cross-reactivity or generalized labeling of the cell body. Some cell bodies are highlighted in white. Scale bar: 5μm.

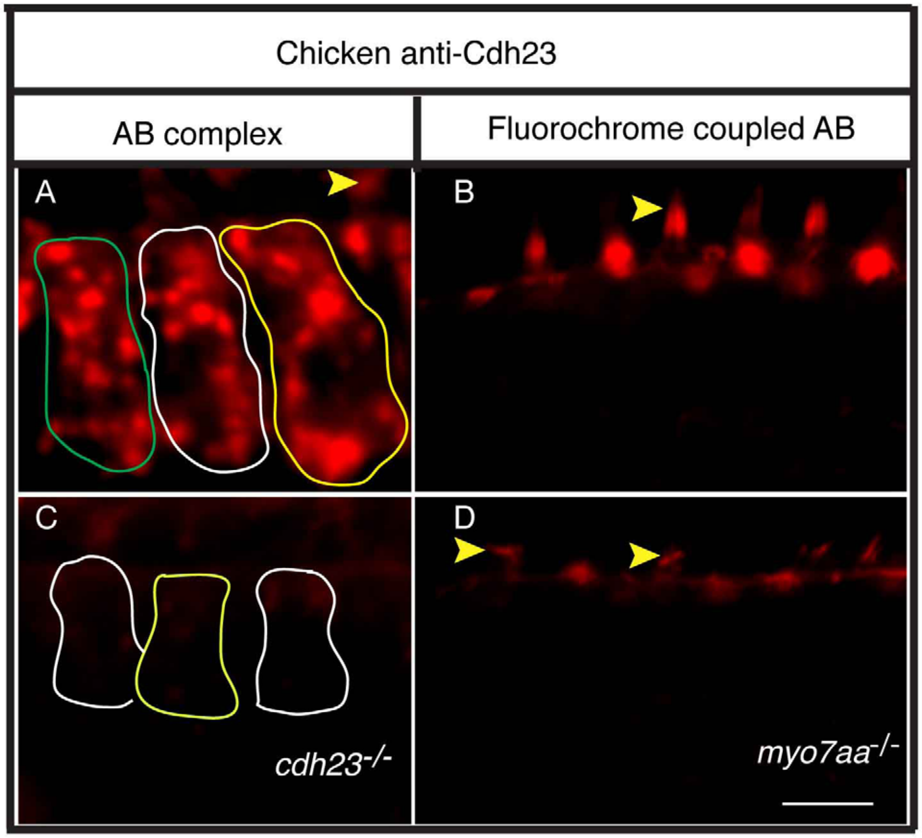

Fig. S5. Cdh23 can reach the stereocilia in *myo7aa* mutants. (A,C) Specificity test for chicken anti-Cdh23 in wild-type sibling (A) and *cdh23*^{tj264a} zebrafish mutant (C) using the AB complex detection method. A reminiscent weak signal is observed in the *cdh23* mutant. This indicates that the *tj264a* mutation does not affect all cadherin 23 isoforms. (B,C) Cdh23 can be detected in the stereocilia of wild-type sibling (B) and *myo7aa* mutant (C) using a direct detection method.. In the direct detection conditions, quantification of the protein amount cannot be done due to saturation of the photodetector or the overall structure of the mechanoreceptor coupled to the lack of cytoplasmic signal in control experimental conditions. Arrowhead: hair bundle protein localization. Cell bodies are highlighted in different colors. Scale bar: 5μm

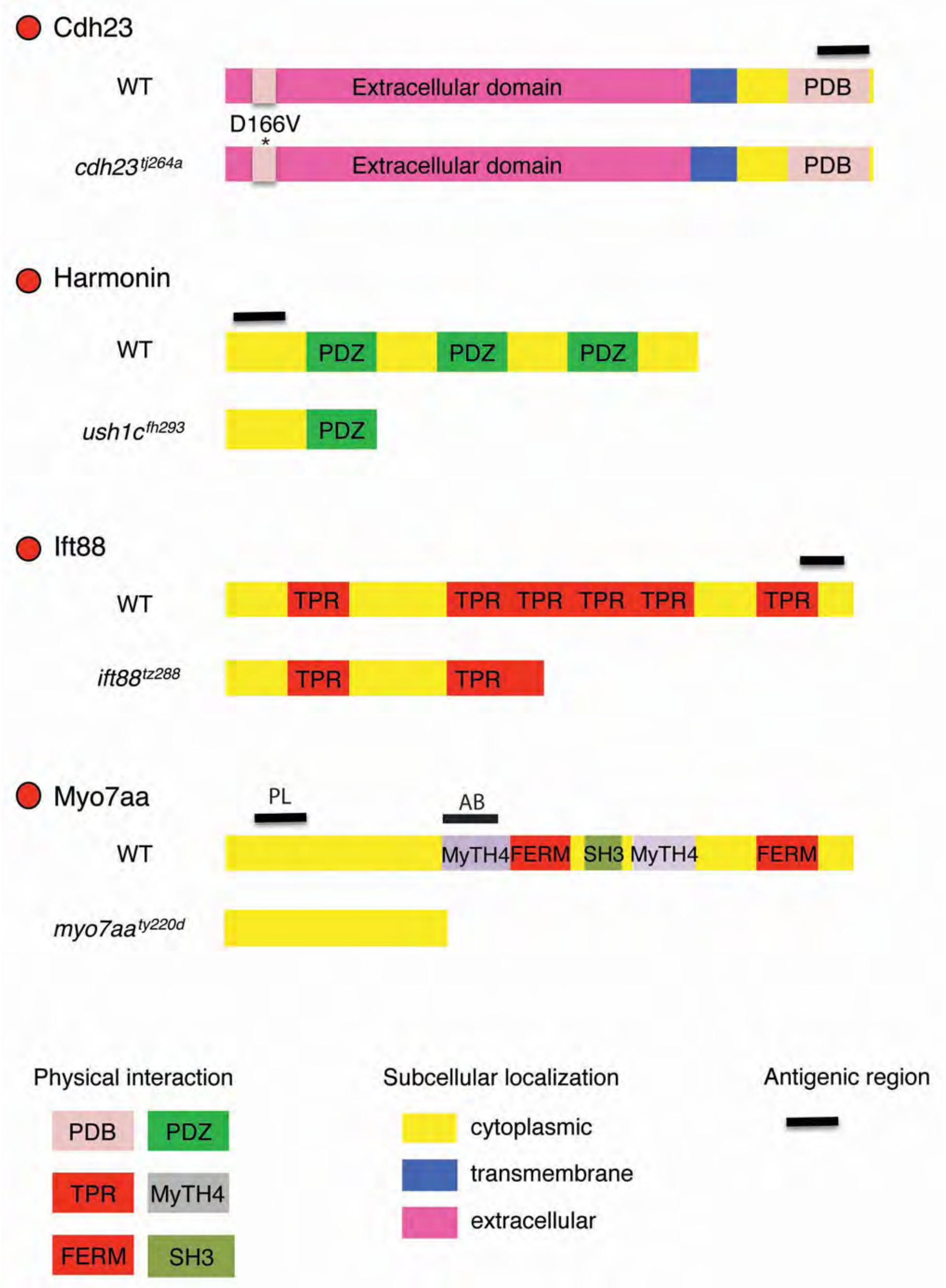

Fig. S6. Schematic representations of protein structures. *cdh23*^{tj264a} is a missense mutation affecting the region of the ectodomain encoded by exon2. *ush1c*^{fh293} is a nonsense mutation that truncates Harmonin just after the first PDZ domain. *ift88*^{tz288b} is a nonsense mutation in exon 11. The encoded product is truncated. *myo7aa*^{ty220d} is a nonsense mutation that truncates Myo7aa in the myosin head region. PDB, PDZ, TPR, MyTH4, FERM, and SH3 are protein domains that mediate physical interactions. Antibody recognition sites are shown by horizontal black bars. For *myo7aa*, recognition sites of the antibodies used for proximity labeling, PL, and antibody labeling, AB, are indicated. The protein lengths are not drawn to scale.

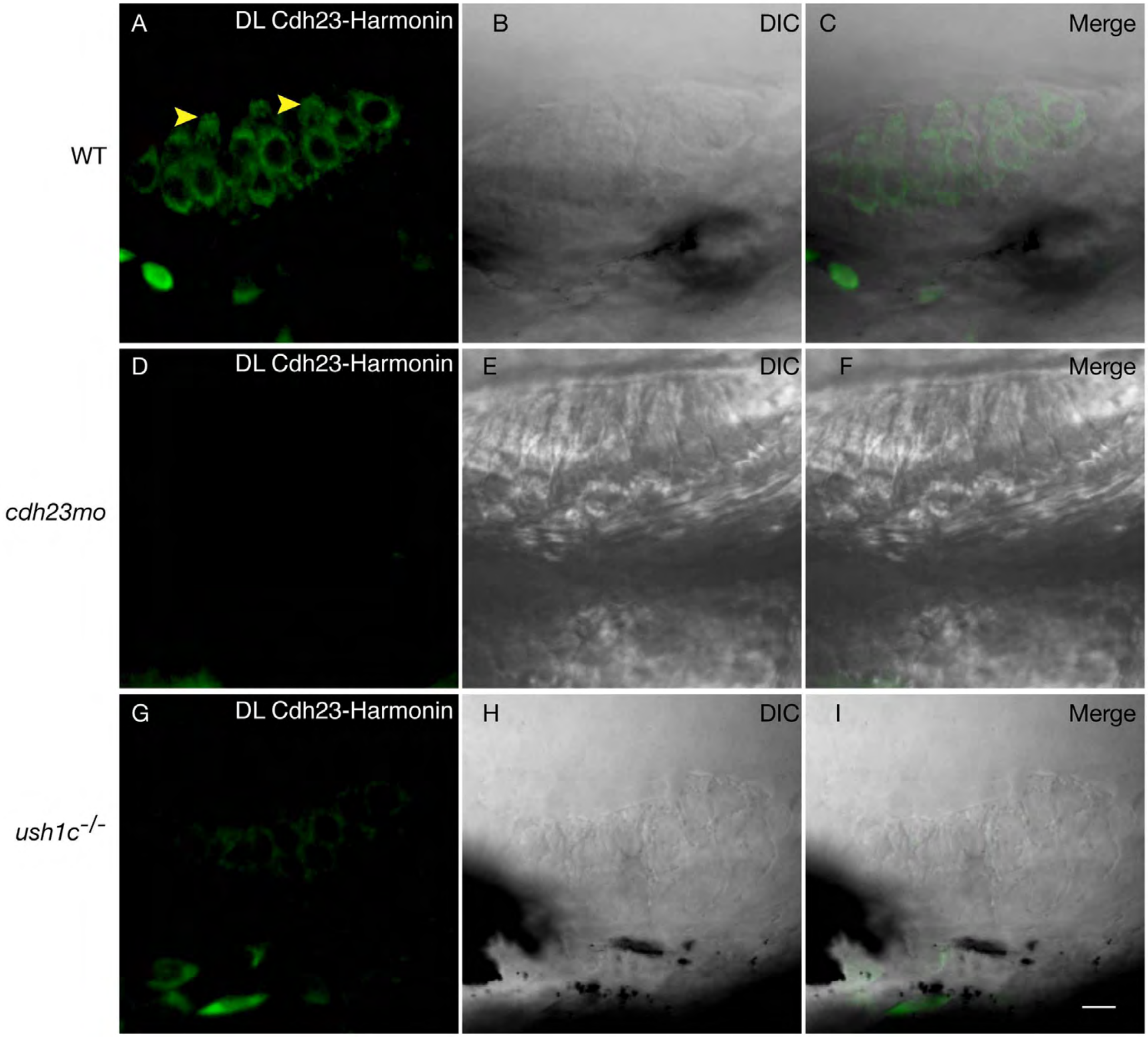

Fig. S7. Proximity labeling of protein pairs is specific. Proximity labeling of Harmonin and Cdh23. (A) In wild-type anterior macula, Harmonin and Cdh23 are in close proximity in hair cell bodies. Note the apical localization of the signal (yellow arrowheads). (B,C): DIC channel and merge of panels A and B. (D-I) Negative controls. No significant signal was obtained when Cdh23 expression was knocked down by injection of *cdh23* morpholinos (D) or in *ush1c* mutants (G). (E,H) DIC channel. (F,I) Merge of D and E, and H and I respectively. WT: wild-type; MO: morpholino. Scale bar: 5 µm.

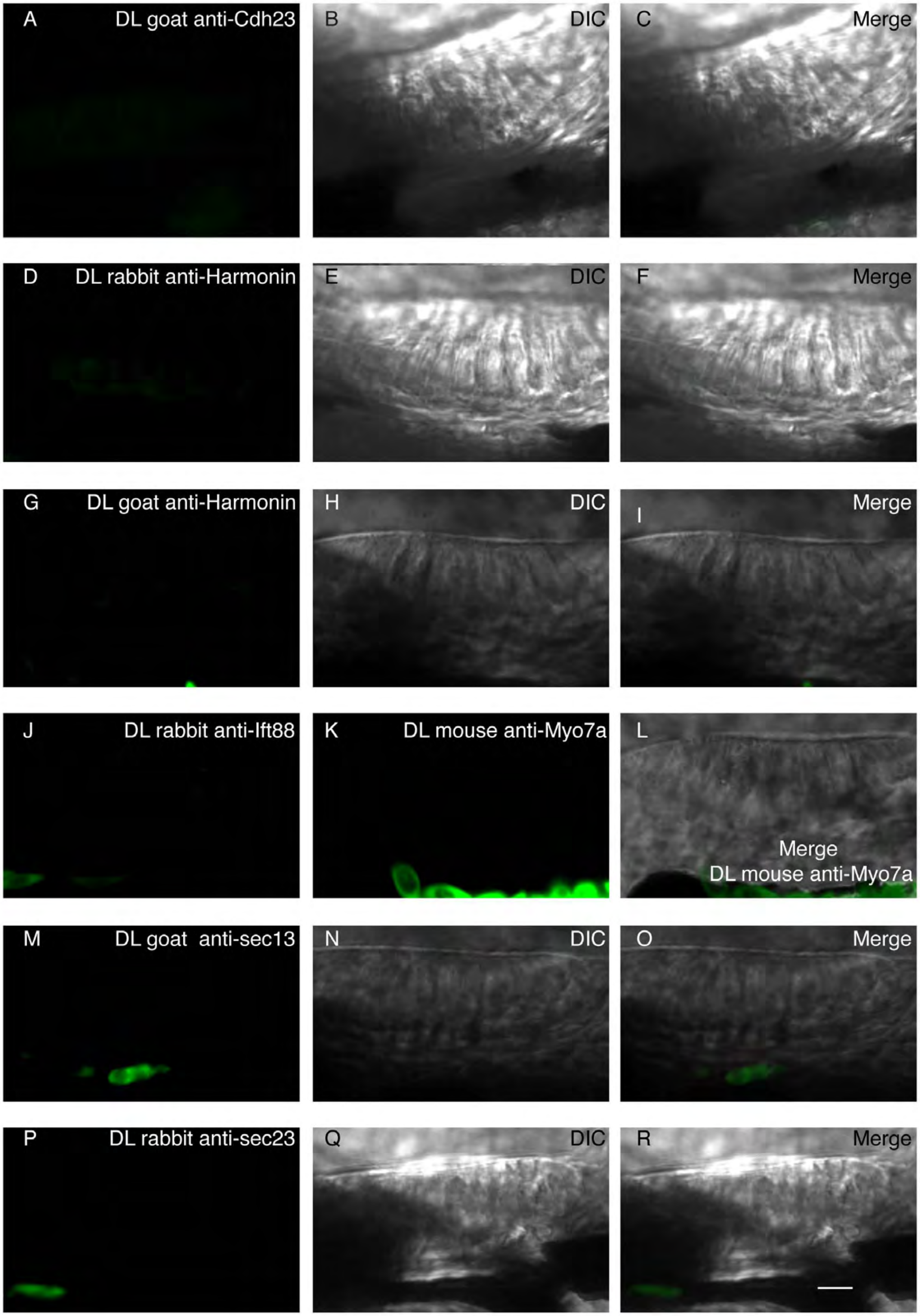

Fig. S8. Proximity ligation control: Assay of nonspecific binding between proximity ligation probes in the absence of one of the primary antibodies during the reaction in WT conditions. (A,D,G,J,K,M,P) The proximity ligation reaction was performed with only one primary antibody: Goat anti-Cdh23 (A), rabbit anti-Harmonin (D), goat anti-Harmonin (G), rabbit anti-Ift88 (J), mouse anti-Myo7a (K), goat anti-Sec13 (M), or rabbit anti-Sec23 (P). (B,E,H,N,Q) single DIC channel showing a lateral view of the anterior macula. (C,F,I,L,O,R) Merged panels. Nonspecific signal was detected when only one primary antibody was added to the reaction. Scale bar: 5μm.

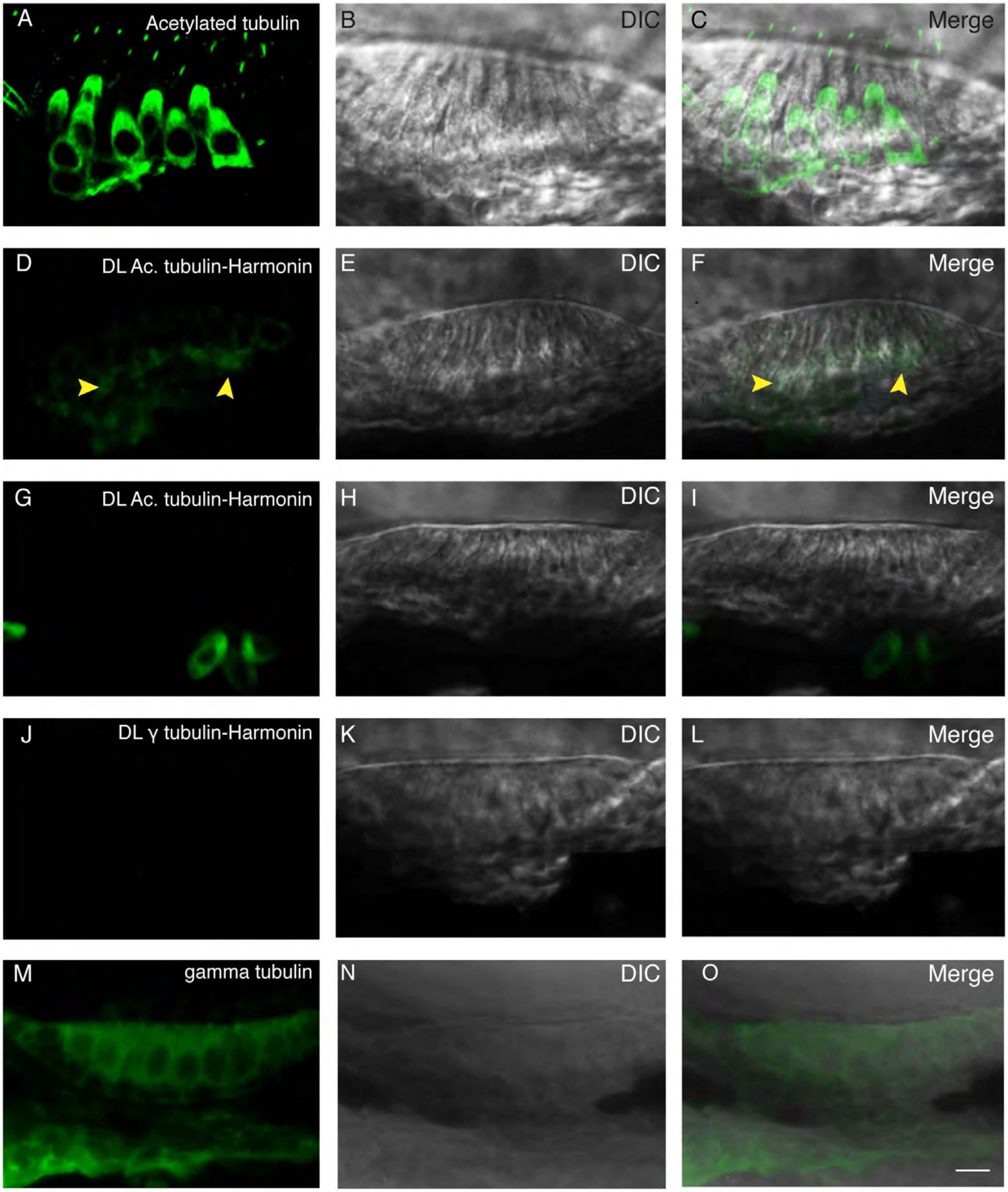

Fig. S9. Proximity ligation assay indicates close physical proximity at the subcellular level. (A) Immunolabeling of acetylated tubulin. Acetylated tubulin fills up the cytoplasm and labels the kinocilium. (B) DIC channel. (C) Merge of panels A and B. (D) Proximity ligation assay of acetylated tubulin and Harmonin results in a restricted signal localized in the basal region of the hair cell, as indicated by the yellow arrowheads. (E) DIC channel. (F) Merge of panels D and E. (G) Proximity ligation assay of acetylated tubulin and Harmonin in *ush1c* mutant results in the absence of signal. (H) DIC channel. (I) merge of panels G and H. (J) Proximity ligation reaction of gamma tubulin and Harmonin resulted in no signal. (K) DIC channel. (L) Merge of panels J and K. (M) Immunolabeling of gamma tubulin showing a nonspecific signal filling the cytoplasm. (N) DIC channel. O: merge of panels M and N. Scale bar: 5μm

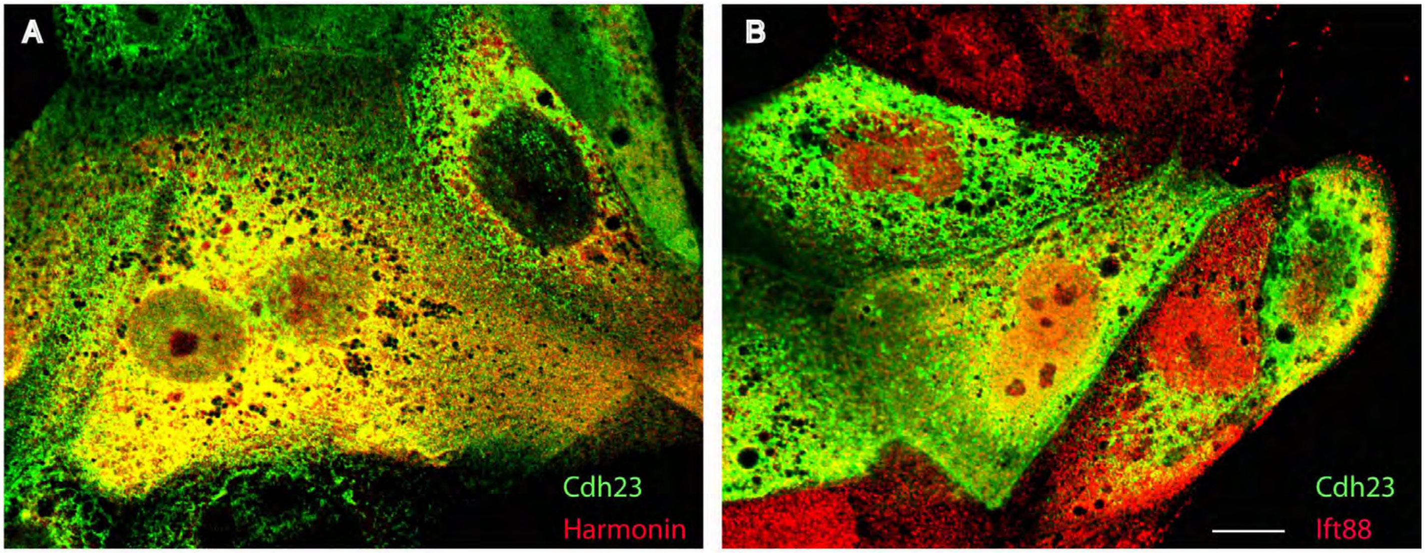

Fig. S10. Subcellular colocalization of Harmonin, Cadherin23 and Ift88 in COS cells. (A) Immunolabeling of mbn-Cdh23-GFP (green) and Harmonin-HA (red). (B) Immunolabeling of mbn-Cdh23-GFP (green) and Ift88-HA (red). Scale bar: 5 µm.

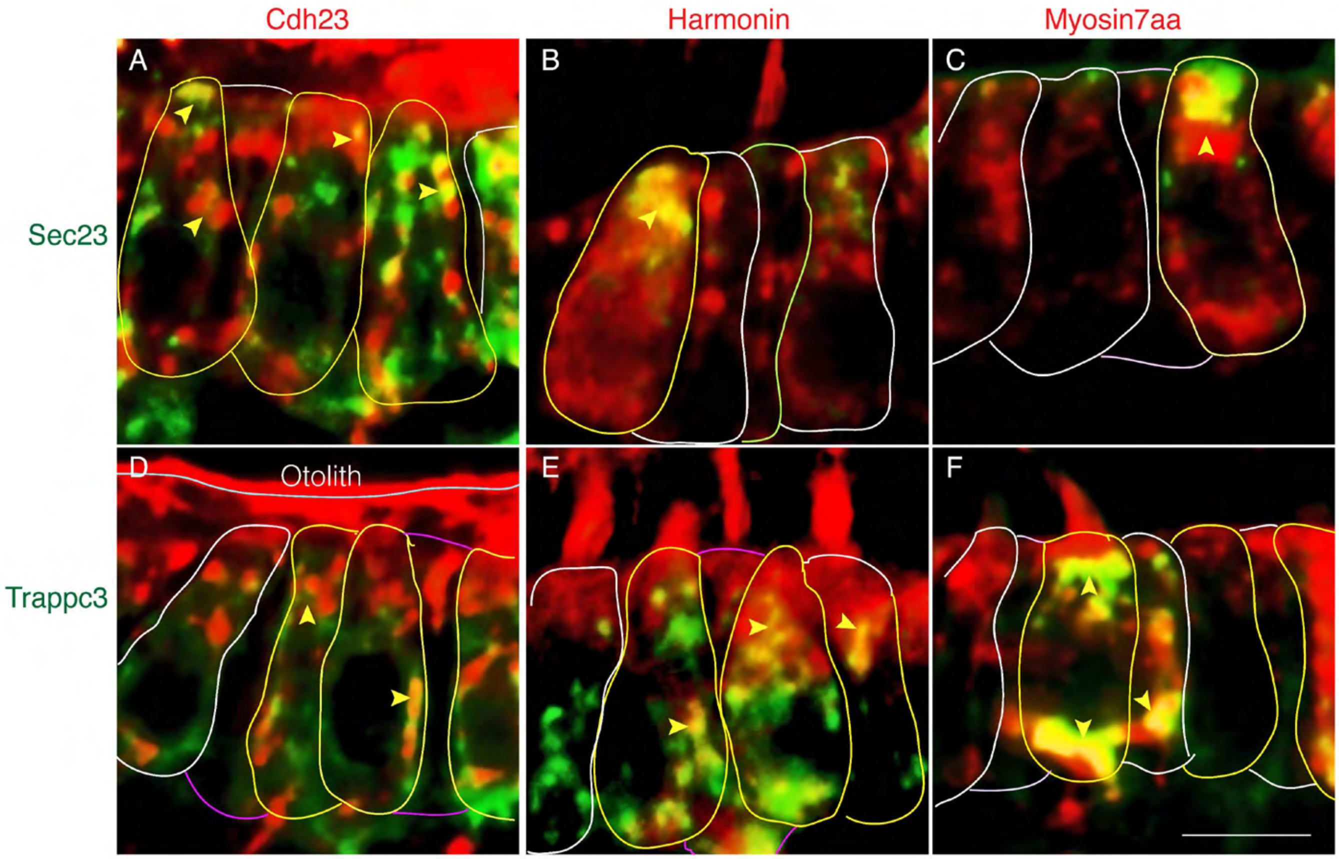

Fig. S11. USH1 proteins colocalize with Sec23 and Trappc3. (A-C) Colocalization of Sec23 and Cdh23 (A), Sec23 and Harmonin (B), Sec23 and Myo7aa (C) in wild-type sibling. (D-F) Colocalization of Trappc3 and Cdh23. (D), Trappc3 and Harmonin (E), Trappc3 and Myo7aa (F) in wild-type sibling. Cell bodies are outlined. Yellow arrowheads indicate colocalization signal in the cell bodies. Scale bar: 5 µm.

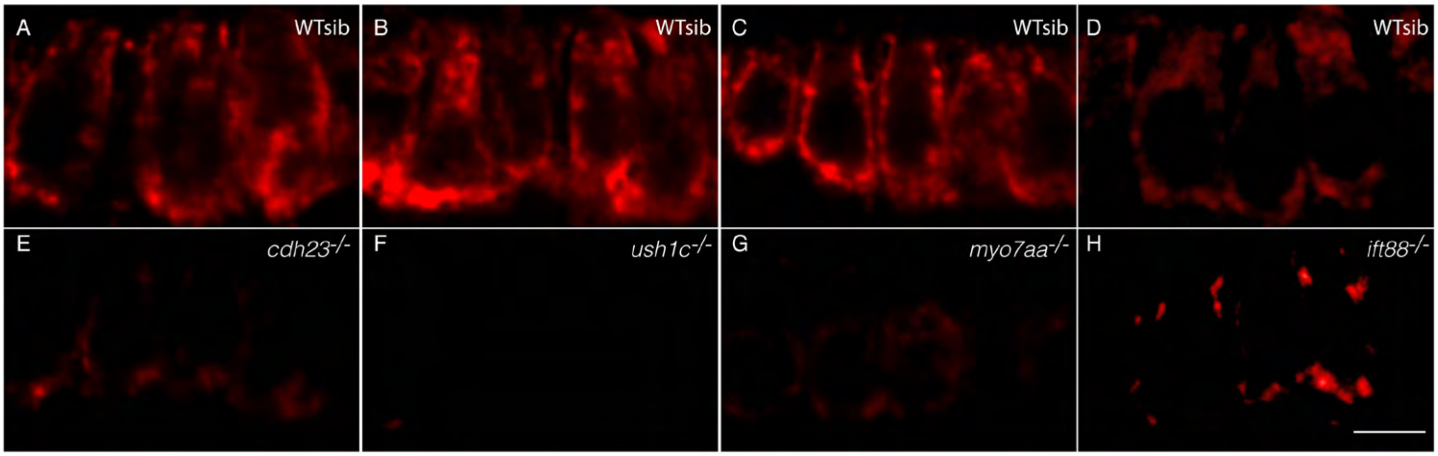

Fig. S12. Loss of ERGIC in *ush1* and *ift88* mutants. (A-D) Wild-type siblings (WT). (E) *cdh23* mutant. (F) *ush1c* mutant. (G) *myo7aa* mutant. (H) *ift88* mutant. Loss of Trappc3 immunolabeling indicates a severe defect during the secretory process in *ush1* and *ift88* mutants. Scale bar: 5 µm

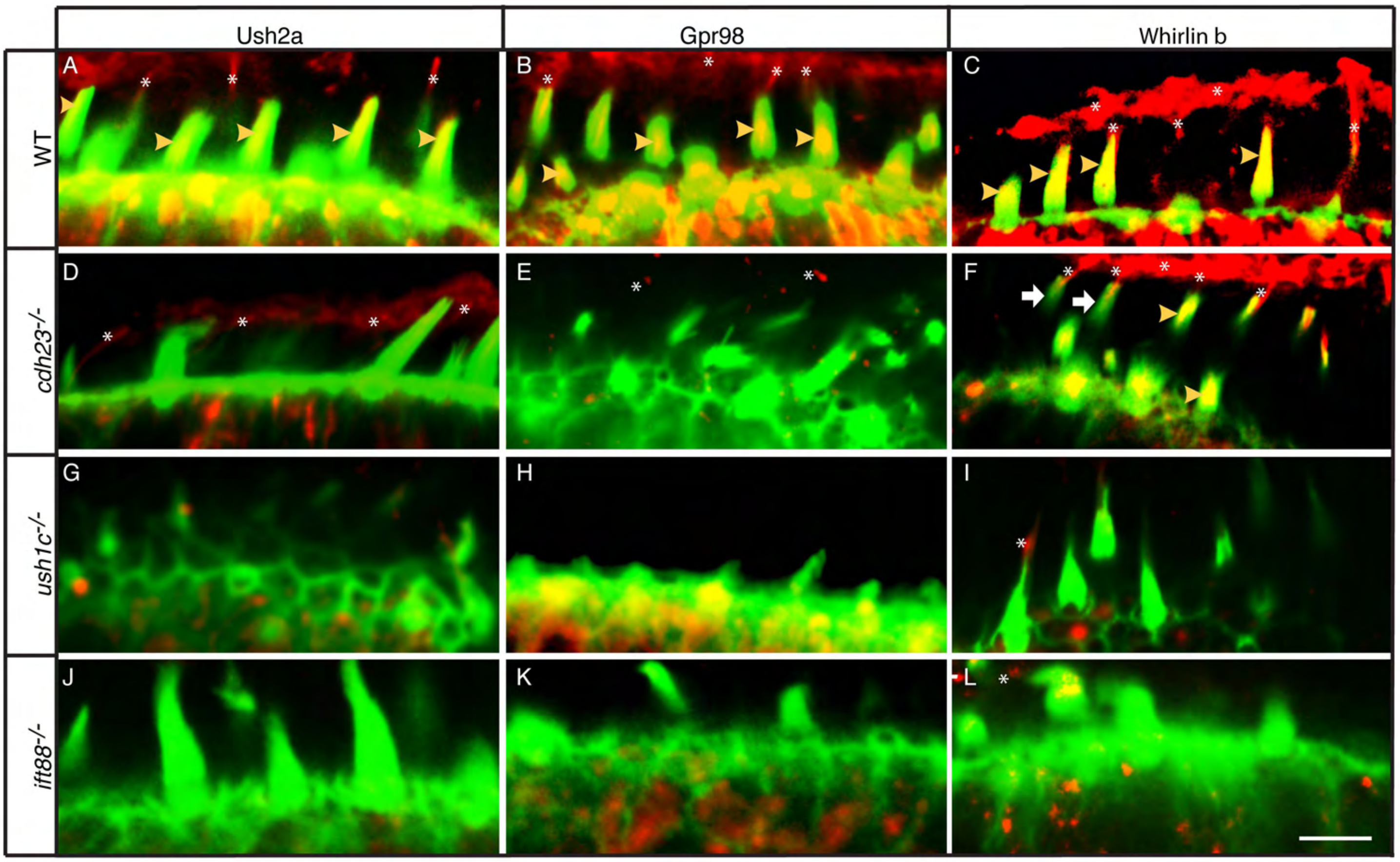

Fig. S13. Hair bundle localization of USH2 Proteins is compromised in *cdh23*, *ush1c*, and *ift88* mutants. (A-L) Phalloidin labeling (green) and immunolabeling (red) of USH2 proteins in (A-C) wild-type siblings or (D-F) *cdh23*, (G-I) *ush1c*, or (J-L) *ift88* mutants with antibodies against Ush2a (A,D,G,J), Gpr98 (B,E,H,K), or Whirlinb (C,F,I,L). All images are single confocal sections at the level of the mechanoreceptor. Yellow arrowheads indicate stereociliary localization of USH2 protein. Asterisks indicate kinociliary localization of USH2 protein. White arrows (F) indicate hair bundles lacking Whirlinb in the *cdh23* mutant. Scale bar: 5 μm.

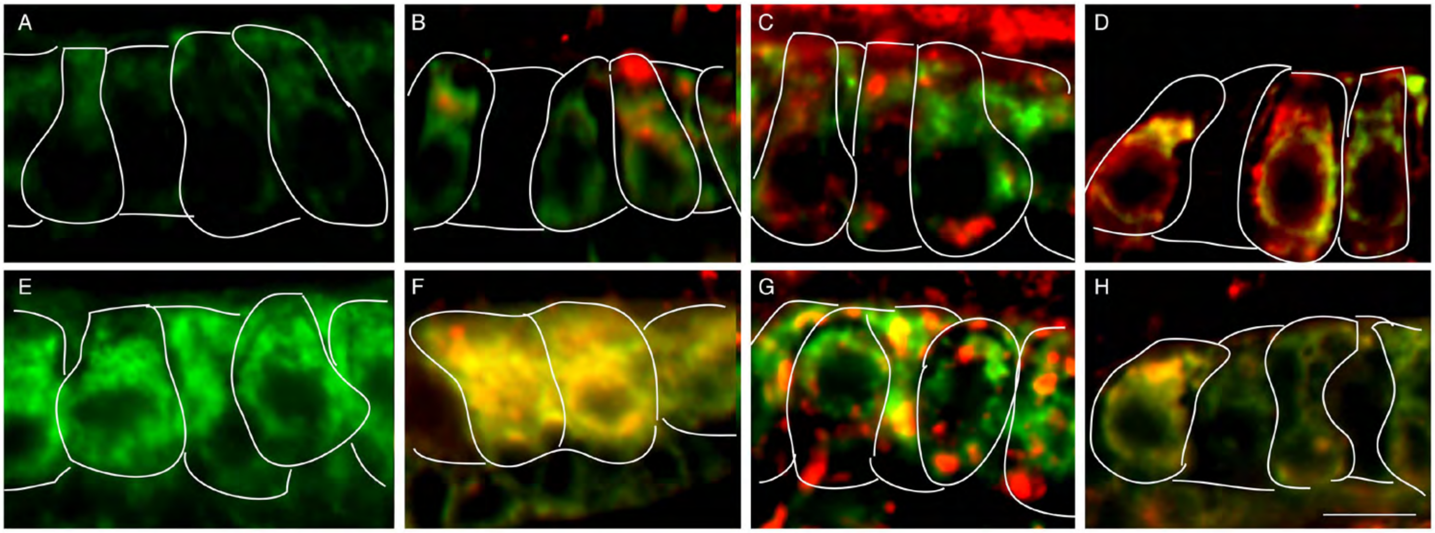

Fig S14. USH2 proteins accumulate in the ER in *ush1c* mutants. (A-D) Wild-type siblings. (E,F) *ush1c* mutants. (A,E) KDEL, a marker of the ER, is expanded in *ush1c* mutants. (B-D,F-H) USH2 proteins are stuck at the level of the ER in *ush1c* mutants. (B,F) Colocalization of Ush2a and KDEL. (C,G) Colocalization of Gpr98 and KDEL. (D,H) Colocalization Whirlinb and KDEL. USH2 proteins can be detected at low levels in the region of the ER (B-D). Colocalization signal between USH2 proteins and KDEL is remarkably increased in *ush1c* mutants. Some cell bodies are outlined. Scale bar: 5µm

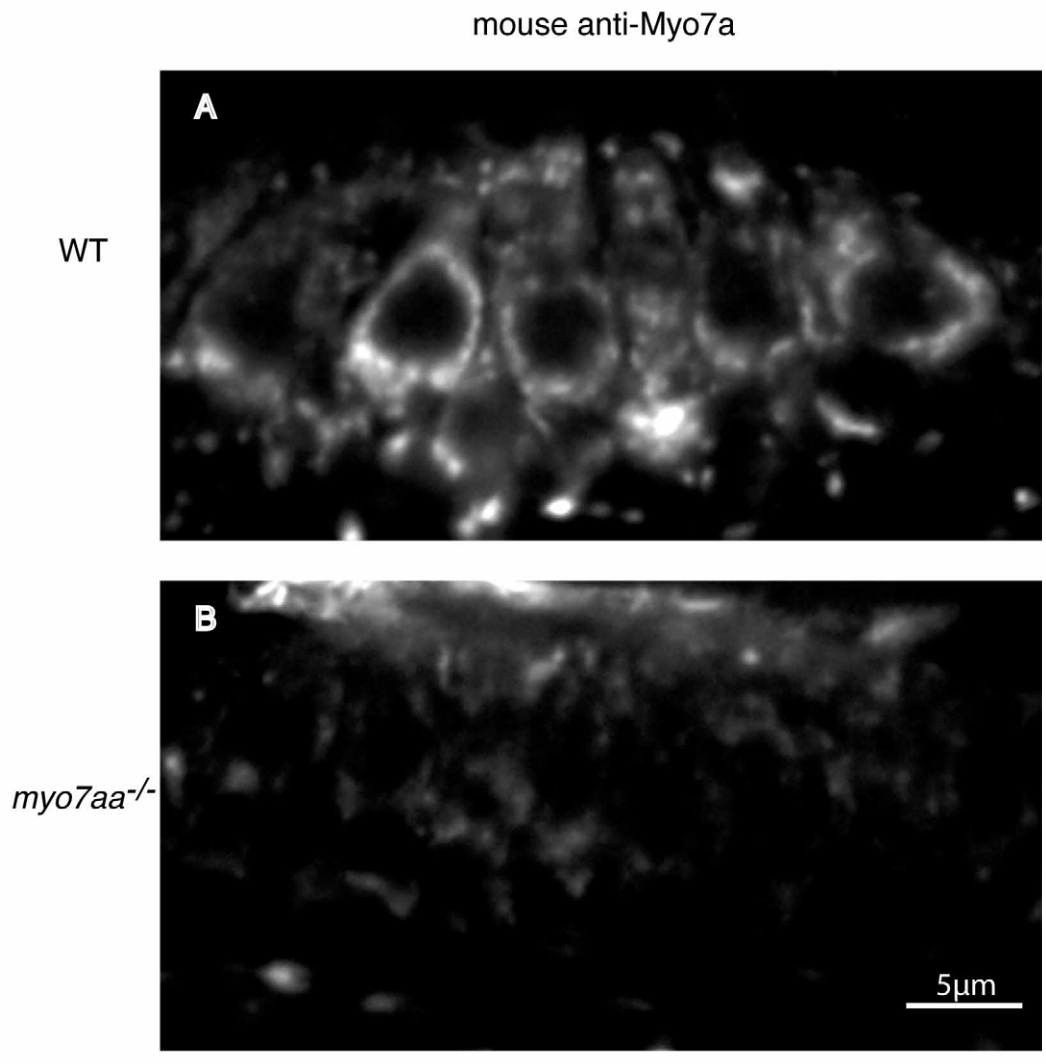

Fig. S15. Mouse anti-Myo7a cross-reacts with zebrafish Myo7aa. (A) Antibody labeling in wild-type zebrafish anterior macula hair cells. (B) Labeling was greatly reduced in *myo7aa* mutants. WT: wild-type. Scale bar: 5 µm.
